# Metabolic fate of pregnene-based steroids in the lactonization pathway of multifunctional strain *Penicillium lanosocoeruleum*

**DOI:** 10.1186/s12934-018-0948-1

**Published:** 2018-06-26

**Authors:** Alina Świzdor, Anna Panek, Paulina Ostrowska

**Affiliations:** Department of Chemistry, Wrocław University of Environmental and Life Sciences, Norwida, 25, 50-375 Wrocław, Poland

**Keywords:** Steroid Baeyer–Villiger oxidation, Steroidal lactone, Biotransformation, Epoxide opening, *Penicillium lanosocoeruleum*, 16-dehydropregnenolone, 16α,17α-epoxypregnenolone

## Abstract

**Background:**

Metabolic activities of microorganisms to modify the chemical structures of organic compounds became an effective tool for the production of high-valued steroidal drugs or their precursors. Currently research efforts in production of steroids of pharmaceutical interest are focused on either optimization of existing processes or identification of novel potentially useful bioconversions. Previous studies demonstrated that *P. lanosocoeruleum* KCH 3012 metabolizes androstanes to the corresponding lactones with high yield. In order to explore more thoroughly the factors determining steroid metabolism by this organism, the current study was initiated to delineate the specificity of this fungus with respect to the cleavage of steroid side chain of progesterone and pregnenolone The effect of substituents at C-16 in 16-dehydropregnenolone, 16α,17α-epoxy-pregnenolone and 16α-methoxy-pregnenolone on the pattern of metabolic processing of these steroids was also investigated.

**Results and discussion:**

All of the analogues tested (except the last of the listed) in multi-step transformations underwent the Baeyer–Villiger oxidation to their δ-d-lactones. The activity of 3β-HSD was a factor affecting the composition of the product mixtures. 16α,17α-epoxy-pregnenolone underwent a rare epoxide opening with retention stereochemistry to give four 16α-hydroxy-lactones. Apart from oxidative transformations, a reductive pathway was revealed with the unique hydrogenation of 5-ene double bond leading to the formation of 3β,16α-dihydroxy-17a-oxa-d-homo-5α-androstan-17-one. 16α-Methoxy-pregnenolone was transformed to the 20(*R*)-alcohol with no further conversion.

**Conclusions:**

This work clearly demonstrated that *P. lanosocoeruleum* KCH 3012 has great multi-functional catalytic properties towards the pregnane-type steroids. Studies have highlighted that a slight modification of the d-ring of substrates may control metabolic fate either into the lactonization or reductive and oxidative pathways. Possibility of epoxide opening by enzymes from this microorganism affords a unique opportunity for generation of novel bioactive steroids.

**Electronic supplementary material:**

The online version of this article (10.1186/s12934-018-0948-1) contains supplementary material, which is available to authorized users.

## Background

Steroids are an important class of natural compounds, ubiquitous in nature and playing crucial functions for metabolism. Their physiological activity depends on the structure, primarily location and stereochemistry of the functional groups attached to the steroid nucleus. A variety of steroid compounds are widely used as anti-inflammatory, anti-allergic, immunosuppressive, anabolic, diuretic, progestational and contraceptive agents. They have also been used for the prevention of coronary heart disease, for the treatment of adrenal insufficiencies and some types of cancers [1]. Structural derivatization of naturally occurring steroid systems can modify their activity profile. Design of new, efficient methods of selective transformations of steroids is a serious challenge because of diverse, and often difficult to predict, reactivity of steroids, which usually are complex, multifunctional chemical species. Carrying out a series of stereospecific reactions occurring selectively is made possible by biotransformations [1–3]. The strongest impulse to use microbial transformations is due to an unusual diversity of enzymes produced by microbes and convenient process conditions. In recent years, Baeyer–Villiger oxidation (BVO) became object of growing interest considering the fact that the result of this reactions can provide steroids with anticancer and anti-androgenic properties.

BVO is an oxidation of ketones, in which cleavage of one of the C–CO–C bonds occurs with simultaneous insertion of an oxygen atom. The products of oxidation of acyclic ketones are esters, while cyclic ketones yield lactones. Oxidative factors employed for this reaction are most often hazardous peroxyacids or hydrogen peroxide. Toxicity and instability of these oxidizers, their explosive tendencies (particularly important in conjunction with flammable solvents in the industrial processes), and lack of selectivity, make chemical synthesis employing BVO seriously limited. In case the substrate is a compound containing other functions vulnerable to oxidation, an important issue is formation of byproducts (e.g. peroxyacids are able to oxidize also double bonds). The enzymes performing the BVO belong to the group of so-called Baeyer–Villiger monooxygenases (BVMOs). It is a class of oxidoreductases using atmospheric oxygen as a “green and free oxidant”. Steroidal BVMOs catalyze mainly oxidation of ketones bound to ring D, i.e. at C-17 and/or C-20 carbonyl groups. Although research on BVO of steroids has long history [4], the substrates of transformations were mostly 4-en-3-keto systems. Interest in this group of steroids was evoked by high antitumor and anti-androgen activity of testolactone (1-dehydrotestololactone). Testolactone as an inhibitor of steroid aromatase [5] is used in the treatment of advanced stages of breast cancer and some symptoms of premature puberty [6]. Steroidal lactones can inhibit activity of steroidal 5α-reductase, and, through interruption of testosterone conversion to 5α-dihydrotestosterone, can be efficient medicines for androgen-dependent syndromes, i.e. benign prostatic hyperplasia and prostate cancer, acne or male pattern baldness [7]. Research on oxidative metabolism of 3β-hydroxy-5-ene steroids has only sped up in the recent years.

Although steroidal BVMO activity was found in many fungi, only two enzymes from bacteria and lower eukaryotes have been characterized until now [8, 9] and whole-cell transformation processes are still the method of choice. Genome of a strain *Penicillium lanosocoeruleum* ATCC 48919, closely related to the one used in the current study, has been sequenced in the framework of the US Department of Energy Joint Genome Institute studies. Numerous enzymes possibly involved in steroid metabolism in this strain include, among others, 214 putative monooxygenases (however, no BVMOs), 27 putative steroid reductases (including three 3-ketosteroid reductases). The function of these proteins was however assigned only by sequence similarity to fungal enzymes of other species. We are not aware of any report linking the sequence information with enzymatic activity of *P. lanosocoeruleum* towards steroids. In light of the large number of possibly involved enzymes that cannot be readily isolated, we have rather adopted the strategy of considering the fungal cell as a micro-bioreactor, and we concentrated on the ability of a given strain to carry out successful biotransformation.

The ability to oxidize ketosteroids to lactones was detected in fungi of different taxonomic classes, especially: *Aspergillus* [10–13], *Fusarium* [14, 15] and *Penicillium* [16–22]. In many transformations, the formation of lactones from 3β-hydroxy-5-ene steroids was accompanied by modification of A-ring occurring through 3β-hydroxy-steroid dehydrogenase/5-ene-4-ene isomerase (3β-HSD) pathway.

**Fig. 1:**
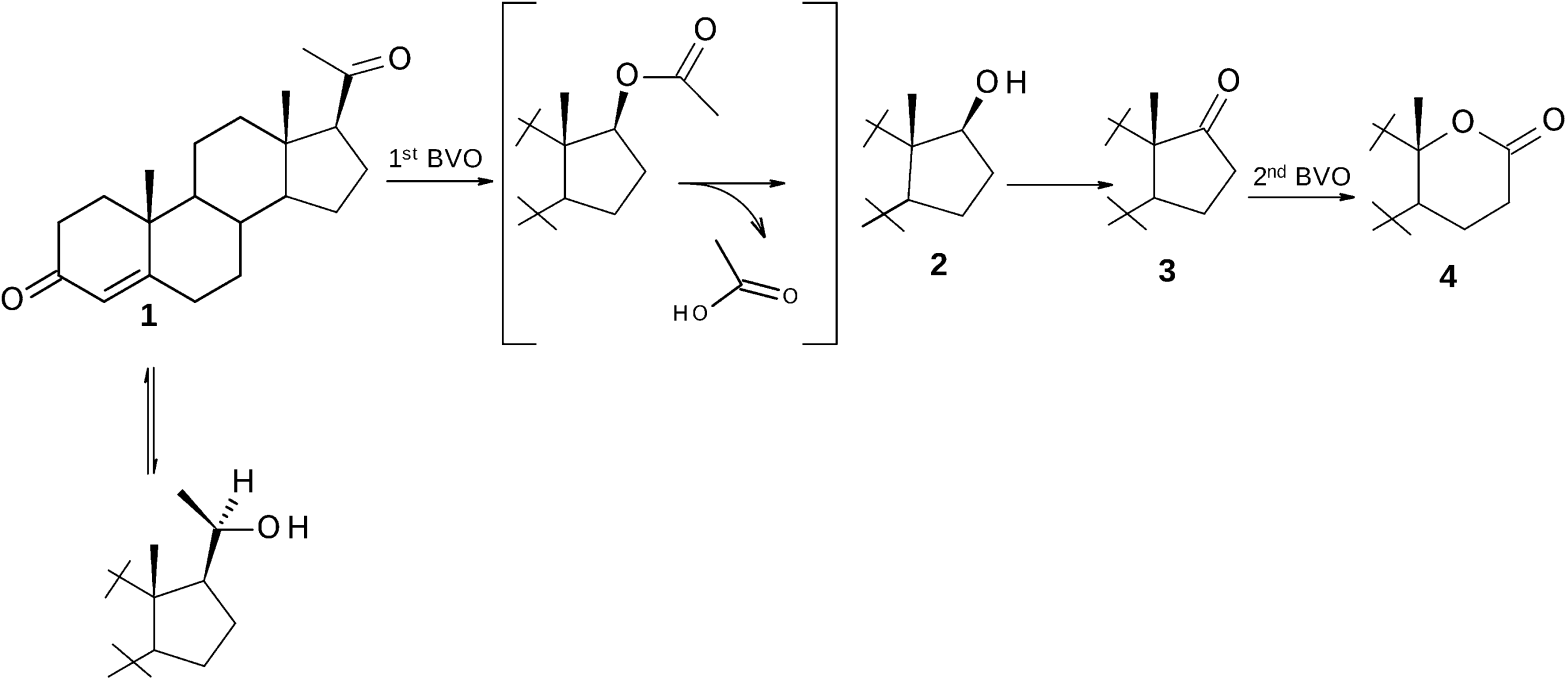
Metabolic pathway of side-chain cleavage of C_21_ steroids

Following the earlier reports [23], it was apparent that cleavage of the pregnane side chain involves four enzymatic reactions: BV oxidation of a substrate to its acetate, hydrolysis of the formed ester to alcohol which is then oxidized to ketone, and oxidative BV lactonization of this ketone to lactone. In transformation of progesterone to testololactone, Sebek and co-workers [16] have proposed a pathway including 20β-hydroxy-4-pregnen-3-one as an intermediate (Fig. 1). In a number of reports the 20-OH analogues of transformed substrates have been isolated [24–26] and it has been suggested that there exists a competitive equilibrium between the reductase forming this alcohol and the oxidase that regenerates the C-20 ketone [16, 27, 28]. A similar relationship was observed in the oxidation of steroidal C-17 ketones by *Beauveria bassiana* [29, 30].

Different microorganisms appeared to exhibit altered specificity with respect to the degradation of steroid side chain. For example, BVMOs from *P. lilacinum* [18], *P. camembertii* [19], *P. simplicissimum* [22], and *F. oxysporum* [15] were able to carry out the degradation of 17β-acetyl side chain of progesterone and pregnenolone, whereas BVMO from *P. citreo*-*viride* [17] and *Beauveria bassiana* [29] were inactive against pregnenolone. Scission at C17–C20 was observed in transformation of cortisone by *A. parasiticus* [13], but cortexolone was transformed to 20(*R*)-alcohol with no further transformation in *A. tamarii KITA* culture [25]. Interestingly, all the above mentioned microorganisms metabolized C-17 ketones to d-homo-lactones.

Previous studies carried out by our group demonstrated that *P. lanosocoeruleum* KCH 3012 metabolizes steroidal 5α-saturated, 4-ene and 5-ene C-17 ketones to the corresponding lactones [21]. In this microorganism steroidal 3β-HSD was active, and as a result dehydroepiandrosterone (DHEA) (a 3β-hydroxy-5-en steroid) was transformed exclusively to testololactone with 3-oxo-4-en functionality in A-ring. In order to explore more thoroughly the factors determining steroid metabolism by this organism, the current study was initiated to delineate the specificity of this microorganism with respect to the cleavage of steroid side chain of basic pregnenes—progesterone and pregnenolone. The effect of substituents at C-16 in pregnenolones (16-dehydropregnenolone, 16α-methoxy-pregnenolone and 16α,17α-epoxy-pregnenolone) on the course of this cleavage and on the general pattern of metabolic processing of these steroids was also investigated.

## Results and discussion

### Products isolated in the course of transformations

#### Biotransformation of progesterone (**1**)

After 72 h of reaction, the substrate was consumed and a single product was isolated. It was testololactone (**4**) (118 mg; 82% mol): ^1^H NMR (300 MHz, CDCl_3_) δ_H_: 1.16 (3H, s, 19-H), 1.35 (3H, s, 18-H), 5.75 (1H, s, 4-H); ^13^C NMR (75 MHz, CDCl_3_): 17.4 (C-19), 19.9 (C-15), 20.1 (C-18), 21.9 (C-11), 28.6 (C-16), 30.5 (C-7), 32.4 (C-6), 33.8 (C-2), 35.5 (C-1), 38.0 (C-8), 38.4 (C-10), 39.1 (C-12), 45.7 (C-14), 52.5 (C-9), 82.7 (C-13), 124.1 (C-4), 169.2 (C-5), 171.1 (C-17), 199.2 (C-3). The spectroscopic data are in agreement with those reported in literature [18].

#### Biotransformation of pregnenolone (**5**)

**Fig. 2:**
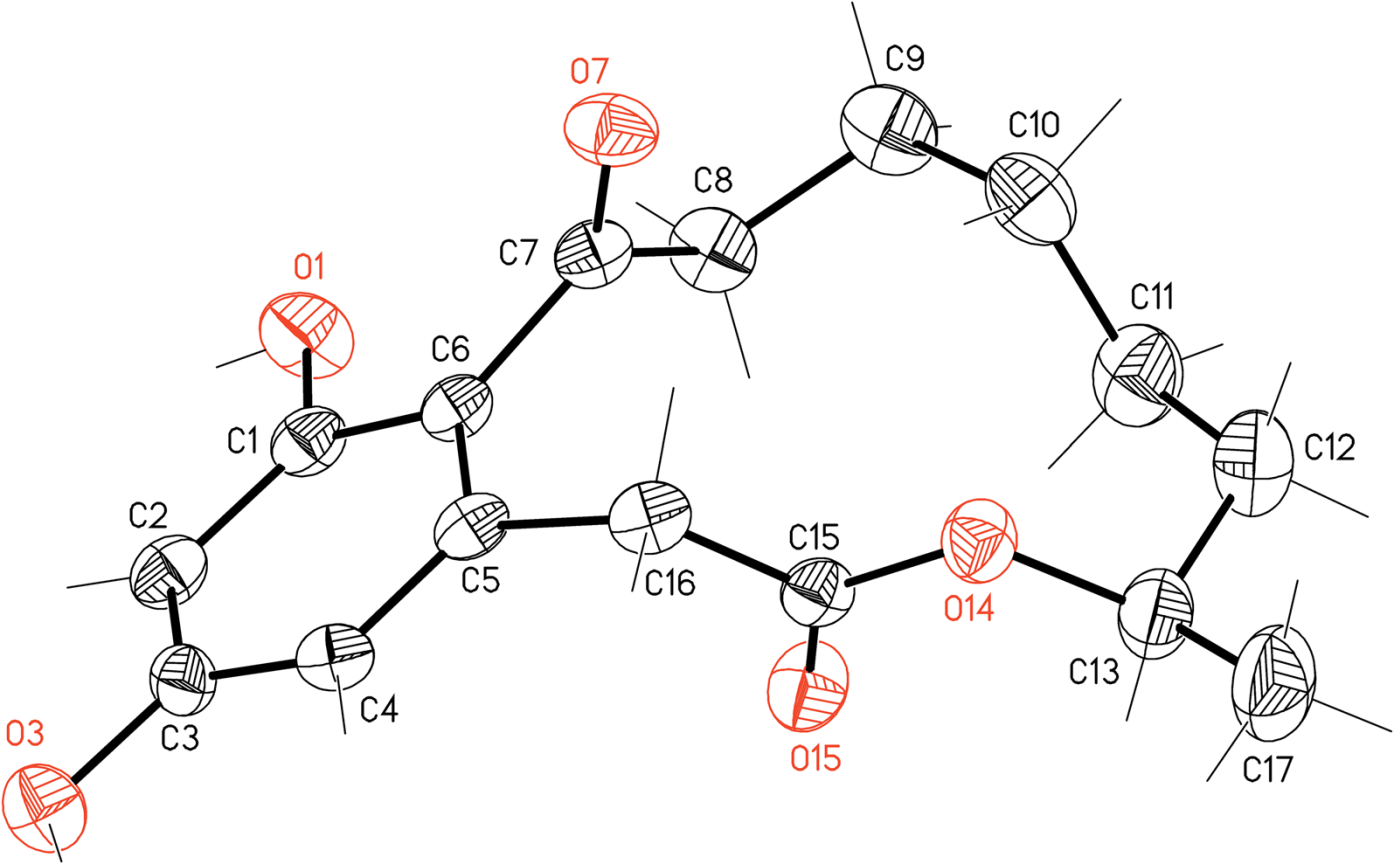
X-ray crystal structure of (*S*)-curvularin (**19**)

After 96 h of fermentation, the following compounds were isolated (% mol): 79 mg (25%) of unreacted pregnenolone (**5**), 40 mg (28%) of testololactone (**4**), and 21 mg of the non-steroidal substance which was identified as (*S*)-curvularin (**19**): colorless crystals, [α]_D_^20^ − 32.1 (c = 1.8 EtOH) (ref.: [α]_D_^20^ − 33.0 (c = 2.0 EtOH) [31]); R_t_ = 3.62 min.; ^1^H NMR (300 MHz, CD_3_OD) δ_H_: 1.16 (3H, d, *J *= 6.3 Hz, 17-CH_3_), 2.70–2.79 (1H, m, 3-H_a_); 3.16–3.25 (1H, m, 3-H_b_), 3.65 (1H, d, *J *= 15.6 Hz, 11-H_a_), 3.86 (1H, d, *J *= 15.6 Hz, 11-H_b_), 4.87–4.97 (1H, m, 8-H), 6.22 (1H, d, *J *= 2.4 Hz, 13-H), 6.25 (1H, d, *J *= 2.4 Hz, 15-H); ^13^C NMR (75 MHz, CD_3_OD): 20.4 (C-17), 23.8 (C-4), 24.9 (C-6), 27.7 (C-5), 33.0 (C-7), 40.4 (C-11), 44.6 (C-3), 73.8 (C-8), 102.7 (C-15), 112.2 (C-13), 120.9 (C-1), 137.2 (C-12), 159.4 (C-14), 161.1 (C-16), 172.8 (C-10), 209.7 (C-2). The spectroscopic data corresponded to those described in literature [32]. The X-ray structure of the product, presented in Fig. 2, is identical with that reported for (*S*)-curvularin [31].

#### Biotransformation of 16-dehydro-pregnenolone (**8**)

After 48 h of fermentation, the following compounds were isolated (% mol): 22 mg (16%) of testololactone (**4**) and 106 mg (73%) of 3β-hydroxy-17a-oxa-d-homo-androst-5-en-17-one (**7**): ^1^H NMR (300 MHz, CDCl_3_) δ_H_: 0.96 (3H, s, 19-H), 1.30 (3H, s, 18-H), 3.53 (1H, tt, *J *= 11.9 Hz, *J *= 5.0, 3α-H), 5.33 (1H, d, *J *= 4.8 Hz, 6-H), ^13^C NMR (75 MHz, CDCl_3_): 19.3 (C-19), 19.9 (C-15), 20.1 (C-18), 21.9 (C-11), 28.8 (C-16), 31.1 (C-8), 31.5 (C-7), 34.4 (C-2), 36.6 (C-10), 36.9 (C-1), 38.9 (C-12), 41.9 (C-4), 46.7 (C-14), 49.0 (C-9), 71.5 (C-3), 83.2 (C-13), 120.6 (C-6), 140.6 (C-5), 171.5 (C-17). The spectroscopic data are in agreement with those reported in literature [18].

#### Biotransformation of 16α,17α-epoxy-pregnenolone (**9**)

After 72 h of fermentation, the following compounds were isolated (% mol): 8 mg (5%) of unreacted substrate **9**, 3β,16α-dihydroxy-17a-oxa-d-homo-androst-5-en-17-one (**10**) (36 mg, 24%), ^1^H NMR (300 MHz, CDCl_3_) δ_H_: 0.97 (3H, s, 19-H), 1.36 (3H, s, 18-H), 3.53 (1H, tt, *J *= 11.4 Hz, *J *= 4.5, 3α-H), 4.46 (1H, dd, *J *= 10.6 Hz, *J *= 4.5, 16β-H), 5.35 (1H, dt, *J *= 5.2, *J *= 1.9 Hz, 6-H), ^13^C NMR (75 MHz, CDCl_3_): 19.2 (C-18), 19.3 (C-19), 21.9 (C-11), 30.6 (C-15), 30.8 (C-7), 31.4 (C-2), 35.2 (C-8), 36.5 (C-10), 36.8 (C-1), 39.0 (C-12), 41.8 (C-4), 46.5 (C-14), 48.6 (C-9), 64.6 (C-16), 71.4 (C-3), 85.1 (C-13), 120.5 (C-6), 140.5 (C-5), 175.2 (C-17); 16α-hydroxy-17a-oxa-d-homo-androst-4-en-3,17-dione (**11**) (62 mg, 41%), ^1^H NMR (300 MHz, CDCl_3_) δ_H_: 1.16 (3H, s, 19-H), 1.39 (3H, s, 18-H), 4.46 (1H, dd, *J *= 10.7 Hz, *J *= 4.6, 16β-H), 5.75 (1H, 4-H), ^13^C NMR (75 MHz, CDCl_3_): 17.3 (C-19), 19.2 (C-18), 21.8 (C-11), 30.2 (C-15), 30.6 (C-7), 32.2 (C-6), 33.7 (C-2), 35.4 (C-1), 38.3 (C-10), 38.6 (C-8), 39.0 (C-12), 45.7 (C-14), 52.1 (C-9), 64.4 (C-16), 84.5 (C-13), 124.2 (C-4), 168.9 (C-5), 174.9 (C-17), 199.0 (C-3); 3β,16α-dihydroxy-17a-oxa-d-homo-5α-androstan-17-one (**12**) (8 mg, 5%), ^1^H NMR (300 MHz, CDCl_3_) δ_H_: 0.77 (3H, s, 19-H), 1.34 (3H, s, 18-H), 3.58–3.63 (1H, m, 3α-H), 4.46 (1H, dd, *J *= 10.7 Hz, *J *= 4.6, 16β-H), ^13^C NMR (75 MHz, CDCl_3_): 12.1 (C-19), 19.3 (C-18), 22.0 (C-11), 28.2 (C-6), 30.4 (C-7), 30.7 (C-15), 31.2 (C-2), 35.4 (C-10), 36.7 (C-1), 37.7 (C-4), 38.6 (C-8), 39.4 (C-12), 44.1 (C-5), 46.4 (C-14), 52.7 (C-9), 64.6 (C-16), 71.0 (C-3), 85.4 (C-13), 175.3 (C-17), and 16α-hydroxy-17a-oxa-d-homo-5α-androstan-3,17-dione (**13**) (8 mg, 5%), ^1^H NMR (300 MHz, CDCl_3_) δ_H_: 0.98 (3H, s, 19-H), 1.36 (3H, s, 18-H), 4.46 (1H, dd, *J *= 10.7 Hz, *J *= 4.6, 16β-H), ^13^C NMR (75 MHz, CDCl_3_): 11.3 (C-19), 19.3 (C-18), 22.1 (C-11), 28.4 (C-6), 30.1 (C-7), 30.7 (C-15), 35.5 (C-10), 37.9 (C-2), 38.1 (C-1), 38.5 (C-8), 39.2 (C-12), 44.2 (C-4), 45.8 (C-14), 46.2 (C-5), 52.2 (C-9), 64.5 (C-16), 85.1 (C-13), 175.1 (C-17), 211.1 (C-3).

#### Biotransformation of 16α-methoxy-pregnenolone (**16**)

After 72 h of fermentation, the following compounds were isolated (% mol): 16α-methoxy-progesterone (**17**) **(**124 mg, 72%) ^1^H NMR (300 MHz, CDCl_3_) δ_H_: 0.66 (3H, s, 18-H), 1.17 (3H, s, 19-H), 2.18 (3H. s, 21-H), 2.54 (1H, d, *J *= 3.0 Hz, 17α-H), 3.20 (3H, s, OCH_3_), 4.32–4.37 (1H, m, 16β-H), 5.74 (1H, s, 4-H). ^13^C NMR (75 MHz, CDCl_3_): 14.6 (C-18), 17.3 (C-19), 20.7 (C-11), 31.7 (C-21), 31.9 (C-7), 32.6 (C-6), 33.9 (C-15), 33.9 (C-2), 35.1 (C-8), 35.6 (C-1), 38.5 (C-10), 38.6 (C-12), 44.3 (C-13), 53.4 (C-9), 53.6 (C-14), 57.2 (C-22); 71.4 (C-17), 81.3 (C-16), 124.0 (C-4), 170.5 (C-5), 199.3 (C-3), 207.9 (C-20), and (20*R*)-20-hydroxy-16α-methoxy-pregn-4-en-3-one (**18**) (26 mg, 15%), ^1^H NMR (300 MHz, CDCl_3_) δ_H_: 0.72 (3H, s, 18-H), 1.18 (3H, s, 19-H), 1.21 (3H, d, *J *= 6.0 Hz, 21-H), 3.32 (3H, s, OCH_3_), 3.86–3.91 (2H, m, 16β-H, 20-H), 5.74 (1H, s, 4-H). ^13^C NMR (75 MHz, CDCl_3_): 13.7 (C-18), 17.3 (C-19), 20.3 (C-11), 23.1 (C-21), 30.6 (C-15), 31.8 (C-7), 32.7 (C-6), 33.9 (C-2), 34.9 (C-8), 35.6 (C-1), 38.4 (C-12), 38.5 (C-10), 42.0 (C-13), 53.1 (C-14), 53.6 (C-9), 57.0 (C-22), 64.6 (C-17), 69.6 (C-20), 86.6 (C-16), 124.0 (C-4), 170.8 (C-5), 199.4 (C-3).

### Structural identification of metabolites

Transformation of progesterone (**1**), pregnenolone (**5**) and 16-dehydro-pregnenolone (**8**) yielded known products: testololactone (**4**) and 3β-hydroxy-17a-oxa-d-homo-androst-5-en-17-one (**7**) which were identified by comparison of their spectroscopic data with the literature values [18] and on the basis of identity of their R_t_ from GC and R_f_ from TLC with standards available in our laboratory. The determination of the structure of the other metabolites was based primarily on their NMR data (see Additional file 1 for the relevant spectra). Thus, all the metabolites of 16α,17α-epoxy-pregnenolone (**9**) were devoid of the side-chain methyl-ketone resonance signals in ^1^H NMR spectrum at δ_H_ 2.02 ppm (21-CH_3_) and in ^13^C NMR spectrum at δ_C_ 26.0 ppm (C-21) and δ_C_ 205.0 ppm (C-20). The absence of these signals fully supported the assumption that removal of the side-chain had occurred. A significant downfield shift in comparison with the substrate was observed for the 18-methyl resonance signals in the ^1^H NMR spectra of these products (from δ_H_ 1.01 ppm for **5** to δ_H_ 1.36 ppm for **9**, δ_H_ 1.39 ppm for **10**, δ_H_ 1.34 ppm for **11**, and δ_H_ 1.36 ppm for **12**). It was consistent with an oxygen atom insertion into the ring-D. This was supported by the ^13^C NMR spectra in which C-13 resonance signal had undergone a downfield shift of ca. 43 ppm (to δ_C_ 85.1 ppm for **10**, to δ_C_ 84.5 ppm for **11**, to δ_C_ 85.4 ppm for **12**, and to δ_C_ 85.1 ppm for **13**). The lactonization in the ring-D of these metabolites, via Baeyer–Villiger oxidation, was confirmed by the appearance of the signal at δ_C_ ca. 175 ppm (C-17). The epoxide 16β-proton (δ_H_ 3.67 ppm) was absent from the spectra of metabolites **10**–**13** indicating epoxide opening. This was supported by a new signal present in the ^1^H NMR spectra of these metabolites at δ_H_ ca.4.46 ppm and in the ^13^C NMR spectra at δ_C_ ca.64.6 ppm which demonstrated the presence of a hydroxyl group. The multiplicity of this signal in ^1^H NMR spectra suggested that it belongs to a proton coupled with only two protons which is consistent with a presence of 16-hydroxyl group. The stereochemistry of this hydroxyl group was further supported by NOESY spectra which showed correlation between H-16β signal and C-18 methyl group and H-15β signals (δ_H_ 2.12 ppm for **10**, δ_H_ 2.05 ppm for **11**, δ_H_ 2.10 ppm for **12**, and δ_H_ 2.05 ppm for **13**). This data led to identification of **10** as 3β,16α-dihydroxy-17a-oxa-d-homo-androst-5-en-17-one. The absence of the 3α-H resonance (tt) from the ^1^H NMR spectrum of the metabolite **11** coupled with an increase in the C-19 methyl resonance signal (Δ 0.12 ppm relative to the substrate **9**) and a shift of the signal of the olefinic proton from δ_H_ 5.32 ppm to δ_H_ 5.75 ppm indicated double bond migration into ring A (between C-4 and C-5) and oxidation of the C-3 alcohol to a ketone. Oxidation of the 3β-OH group was fully supported by the loss of a methine signal at δ_C_ 74.6 ppm in the starting material ^13^C NMR spectrum, being replaced by a new non-protonated signal in the spectrum of the product at δ_C_ 199.0 ppm. Thus, the structure of metabolite **11** was deduced to be 16α-hydroxy-17a-oxa-d-homo-androst-4-en-3,17-dione. The NMR spectra of product **12** revealed that there were two hydroxyl groups (multiplet at δ_H_ 3.58–3.63 ppm and doublet of doublets at 4.46 ppm) and no carbon–carbon double bond. In comparison to the ^13^C NMR spectrum of **10**, the disappearance of resonance signal at δ_C_ 120.5 ppm and δ_C_ 140.5 ppm confirmed hydrogenation of this double bond. This was further supported by a new presence of methine C-5 and methylene C-6 signals at δ_C_ 44.1 ppm and δ_C_ 28.2 ppm, respectively. Hydrogenation resulting in 5α-stereochemistry at A/B rings was determined by chemical shift of C-19 methyl group signal, which was resonating at similar field (δ_C_ 12.1 ppm and δ_H_ 0.77 ppm) when compared with known 3β-hydroxy-17a-oxa-d-homo-5α-androstan-17-one [21]. Therefore, the metabolite **12** was proposed to be 3β,16α-dihydroxy-17a-oxa-d-homo-5α-androstan-17-one. The ^13^C NMR spectrum of **13** was similar to that of **12** with the exception of signals of the A-ring carbons. The absence of the C-3 methine signal at δ_C_ 71.0 ppm and the appearance an additional quaternary carbon signal at δ_C_ 211.1 ppm indicated the oxidation of the C-3 hydroxyl to a carbonyl group. Also, the signal of 3α-proton disappeared in the ^1^H NMR spectrum and the chemical shift of C-19 methyl group signal was resonating at similar field (0.98 ppm) when compared with known 17a-oxa-d-homo-5α-androstan-3,17-dione [21]. This data led to the identification of **13** as 16α-hydroxy-17a-d-homo-5α-androstan-3,17-dione.

The resonance signals in both ^1^H and ^13^C NMR spectra of **17** and **18** suggested changes in the ring A and B of these molecules with respect to the substrate **16**. The absence of the 3α-H multiplet at δ_H_ 3.52 ppm and a downfield shift signal of olefinic proton from δ_H_ 5.34 ppm to δ_H_ 5.74 ppm indicated isomerization of the double bond with its formation between C-4 and C-5 and oxidation of the C-3 alcohol to a ketone. Oxidation of the 3β-OH group was fully supported by the appearance of a new non-protonated signal at δ_C_ ca. 199 ppm. Its position was consistent with the position of a β-carbon conjugated with the carbonyl group. All these observations confirm the formation of 3-oxo-4-en moiety in the obtained products. Thus, metabolite **17** was identified as 16α-methoxyprogesterone. Evidence for identification of (20*R*)-20-hydroxy-16α-methoxy-pregn-4-en-3-one (**18**) was provided by the ^13^C NMR spectrum with loss of the resonance signal at δ_C_ 207.9 ppm for the C-20 ketone and its replacement with a H*C*OH signal at δ_C_ 69.6 ppm. Also, the C-21-methyl signal of **18** underwent an upfield shift (Δ 8.6 ppm) accordingly to the presence of the less electronegative alcohol, and the multiplet visible at δ_H_ 3.89 ppm showed correlation with the carbon C-20 in HSQC spectrum. Additional confirmation of C-20 reduction was the upfield shift (Δ 6.8 ppm) of resonance for C-17. This was coupled with the significant upfield shift (Δ 0.46 ppm) of the 16β-H resonance signal in ^1^H NMR spectrum, and NOESY spectrum showed correlation of 16α-OCH_3_ signal with the proton signal of C-20.

The structure of the non-steroidal compound **19** was determined by 2D NMR experiments and supported by the single crystal X-ray analysis (Fig. 2), which yielded a structure identical with a previous report [31]. The physical data, in particular optical rotation value of **19** were in agreement with those of (*S*)-curvularin published previously [31, 32].

**Fig. 3:**
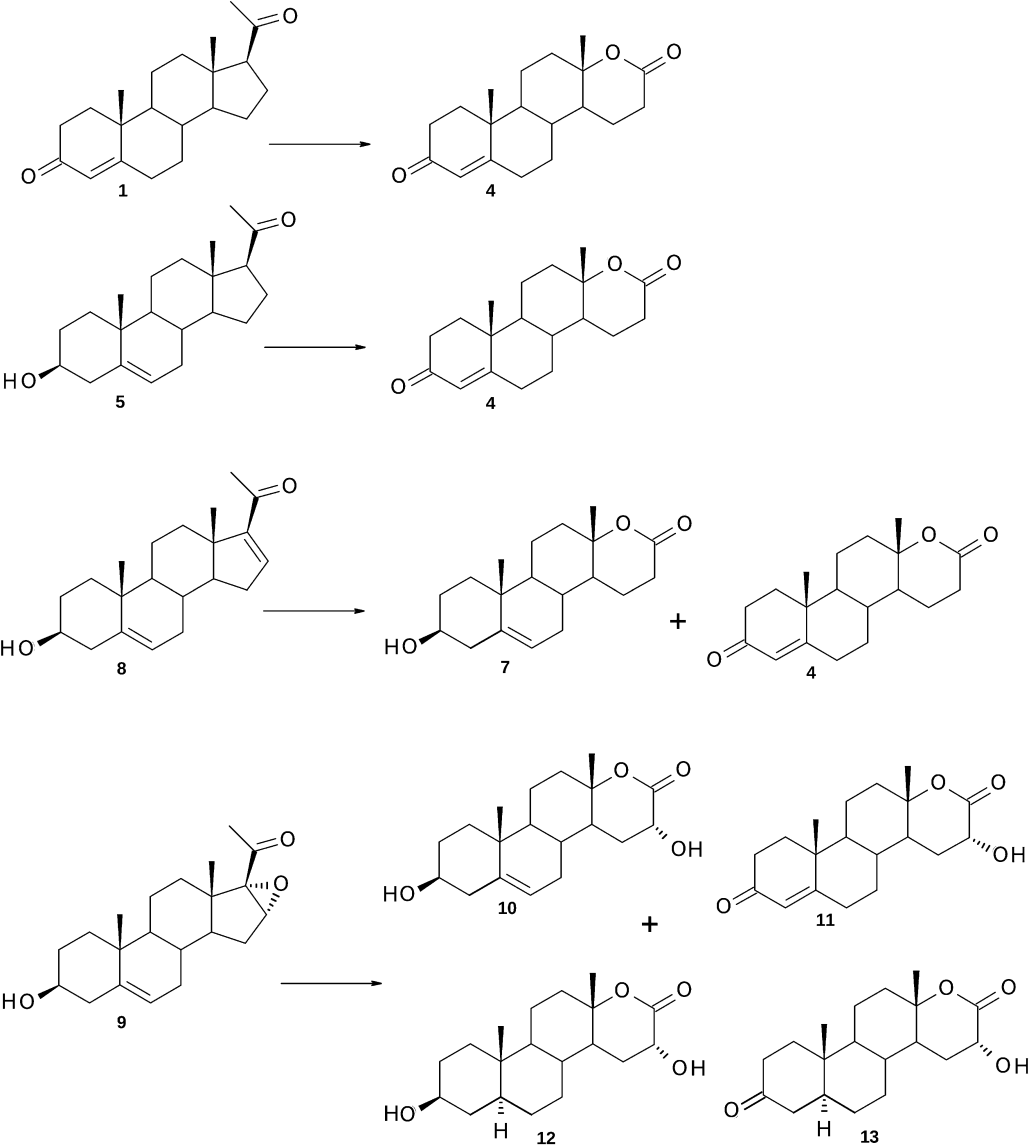
Metabolites isolated following transformation of progesterone (**1**), pregnenolone (**5**), 16-dehydro-pregnenolone (**8**) and 16α,17α-epoxy-pregnenolone (**9**) by *P. lanosocoeruleum*

The results herein presented clearly show that enzymes of *P. lanosocoeruleum* KCH 3012 are involved in the degradation of the C-17β-acetyl side chain of progesterone (**1**), pregnenolone (**5**), 16-dehydro- (**8**) and 16α,17α-epoxy-pregnenolone (**9**) via oxygenative esterification of these 20-ketosteroids and hydrolyze esters into alcohols and acetic acid. The subsequently formed 17-keto steroids could be then oxygenated by the lactonizing enzyme to their respective lactones (Fig. 3). Especially, for progesterone (**1**) the multistep transformation led to afford testololactone (**4**) as a single product isolated with 82% yield. The same metabolite (although with almost three times lower yield) was obtained from pregnenolone (**5**) and, as a minor metabolite, from 16-dehydro-pregnenolone (**8**). The main product of the conversion of **8** was lactone with conserved ring-A of the substrate—3β-hydroxy-17a-oxa-d-homo-androst-5-en-17-one (**7**).

In order to investigate metabolic pathways of these two structurally related compounds (**5** and **8**), we studied composition of mixtures sampled after various transformation periods (Table 1). Their analysis indicated that the first stage of the transformation of pregnenolone (**5**) was the oxidative conversion its 3β-hydroxy-5-ene group to 3-oxo-4-ene system which resulted in progesterone (**1**), the product of its 17β-acetyl chain cleavage—testosterone (**2**), and its subsequent oxidation—androstenedione (**3**). Since the moment of identification this 3-oxo-4-ene C_19_ metabolites, the content of testololactone (**4**)—product of BV oxidation of C-17 ketone significantly increased reaching a maximum at 72 h of the process. Only small amount of DHEA (**6**) and 3β-hydroxy-lactone (**7**) were identified in the reaction mixtures even if the substrate **5** was present throughout the studied period of transformation.

**Table 1:** The time course of the transformation of progesterone, pregnenolone and 16-dehydropregnenolone by *P. lanosocoeruleum*

Substrate	R_t_ (min)	Steroidal compounds identified in the mixture (%)^a^	Time of transformation (h)				
			9	24	48	72	96
Progesterone (**1**)	6.82	Progesterone (**1**)	67	18	6	2	
	4.76	Testosterone (**2**)^b^	26	36	3	–	
	4.54	Androstenedione (**3**)^b^	7	10	5	2	
	8.69	Testololactone (**4**)	–	36	85	88	
Pregnenolone (**5**)	5.57	Pregnenolone (**5**)	75	54	34	28	19
	6.82	Progesterone (**1**)^b^	5	2	3	4	2
	4.76	Testosterone (**2**)^b^	11	13	5	6	–
	4.54	Androstenedione (**3**)^b^	4	3	8	3	3
	3.51	DHEA (**6**)^b^	3	4	2	–	–
	6.91	3β-Hydroxy-17a-oxa-d-homo-androst-5-en-17-one (**7**)^b^	–	2	6	8	5
	8.69	Testololactone (**4**)	2	19	41	44	31
16-Dehydropregnenolone (**8**)	5.22	16-Dehydropregnenolone (**8**)	84	12	–	–	
	6.91	3β-Hydroxy-17a-oxa-d-homo-androst-5-en-17-one (**7**)	10	75	81	70	
	8.69	Testololactone (**4**)	5	13	18	30	

^a^Determined by GC analysis of the crude chloroform extracts

^b^Identified in GC and TLC on the basis of standard

**Fig. 4:**
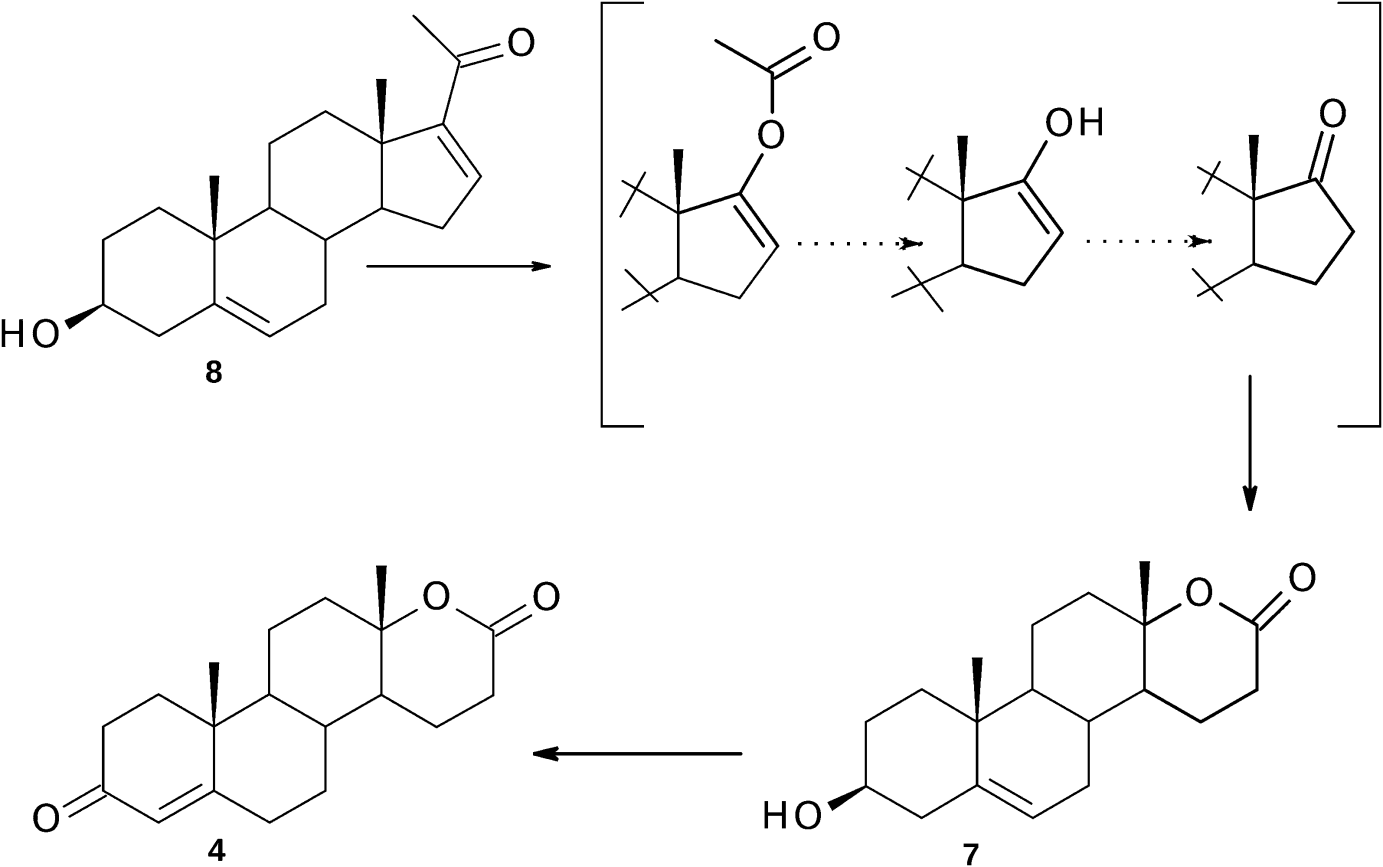
The possible metabolic pathway of 16-dehydro-pregnenolone (**8**) in *P. lanosocoeruleum*

The products of scission of 17β-acetyl group—DHEA and androstenedione were not identified during the transformation of 16-dehydropregnenolone (**8**). Comparison of the course of transformation of both C_21_-20-ketones **5** and **8** in time indicated that 16-dehydro-analog of pregnenolone underwent conversion noticeably faster. Approximately 90% of the incubated **8** was affected by the enzymatic action of the fungus during first 24 h of transformation (Table 1). These reactions were mainly related to the ring-D of molecule. Thus, the oxidation in D-ring of **8** led to enol acetate which could then be rapidly hydrolyzed by an esterase, subsequently undergo rapid non-enzymatic rearrangement to DHEA and further oxidation to the respective lactone (Fig. 4). However, the final metabolites of 16(17)-dehydropregnenolone (**8**) were both lactones—3β-hydroxy-5-ene as well as 3-oxo-4-ene lactone (respectively, **7** and **4**) (Fig. 4). The mixture after 24 h incubation of **8** contained larger percentage of hydroxylactone **7** than testololactone (**4**), whereas after transformation completion the amount of **4** was only twofold lower. Because at the same time the substrate **8** was not identified in the extracts, lactone **4** could be formed only via the conversion of hydroxylactone **7** (3β-HSD activity occurred following d-lactonization).

**Fig. 5:**
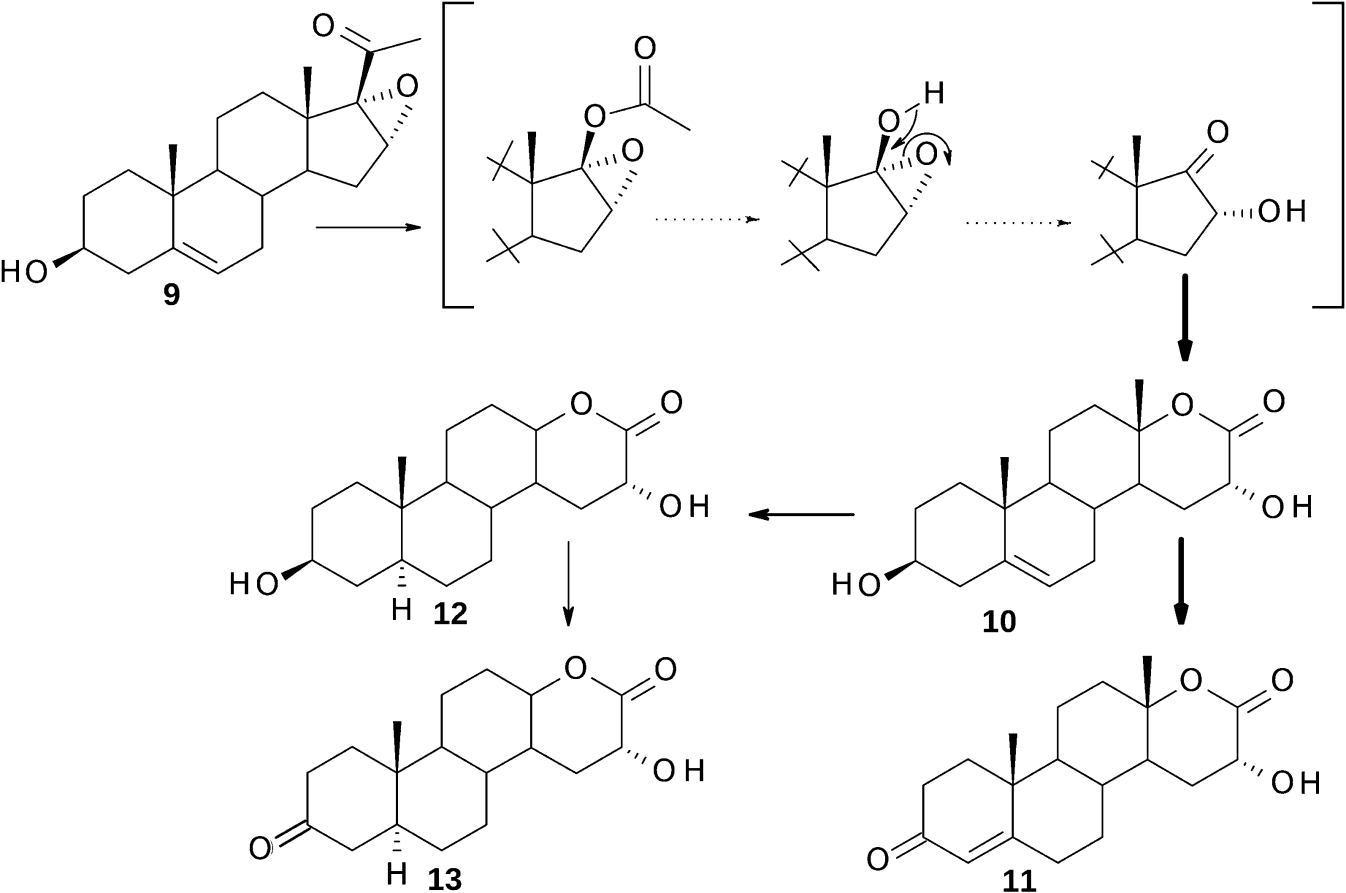
The possible metabolic pathway of 16α,17α-epoxy-pregnenolone (**9**) by *P. lanosocoeruleum*

When 16α,17α-epoxy-pregnenolone (**9**) was incubated with *P. lanosocoeruleum* we obtained mixture of four hydroxylactones. The substrate underwent a rare epoxide opening [25, 33–35] resulting in retention of *alpha* stereochemistry to give 16α-hydroxy metabolites **10**–**13** (Fig. 3). The proposed mechanism of this transformations is outlined in Fig. 5: cleavage at C17–C20 bond gives 16α-hydroxy-17-ketone which is subsequently converted via Baeyer–Villiger oxidation to 3β,16α-dihydroxy-17a-oxa-d-homo-androst-5-en-17-one (**10**). Other metabolites of **9** were mostly products of further transformations of this 16α-hydroxylactone. The observed reactions included the oxidation of hydroxyl group at C-3 to ketone and the subsequent isomerization of double bond C=C from C-5 to C-4 and hydrogenation of C5–C6 double bond. The time experiments indicated that the relative content of **10** was decreasing and 16α-hydroxy-17a-oxa-d-homo-androst-4-en-3,17-dione (**11**) began to dominate in the mixture of metabolites after 72 h of transformation (Table 2). Neither 16α,17α-epoxyprogesterone (**14**) nor 16α-hydroxyandrostenedione (**15**) were identified in any of the reaction mixtures, which suggests that 3β-HSD activity occurred here following ring d-lactonization. This is in contrast to previous studies in which 3β-HSD was active in the presence of a C-17 ketone as in DHEA [21] or a C-17 side chain such as pregnenolone (**5**). It is also interesting to note that the minor metabolites of **9** were 5α-saturated 16α-hydroxylactones (**12** and **13**). Although microbial hydrogenation of 4-en-3-keto steroids to 5-dihydrosteroids using *Penicillium* species has been described [12, 36], there is only one report for the reduction of C5-C6 double bond in ring B in 3β-hydroxy steroids [20].

**Table 2:** The time course of the transformation of 16α,17α-epoxy-pregnenolone and 16α-methoxy-pregnenolone by *P. lanosocoeruleum*

Substrate	R_t_ (min.)	Steroidal compounds identified in the mixture (%)^a^	Time of transformation (h)			
			12	24	48	72
16α,17α-Epoxy-pregnenolone (**9**)	6.38	16α,17α-Epoxy-pregnenolone (**9**)	100	93	19	7
	8.30	3β,16α-Dihydroxy-17a-oxa-d-homo-androst-5-en-17-one (**10**)	–	2	22	26
	8.59	16α-Hydroxy-17a-oxa-d-homo-androst-4-en-3,17-dione (**11**)	–	1.5	31	48
	9.06	3β,16α-Dihydroxy-17a-oxa-d-homo-5α-androstan-17-one (**12**)	–	1	7	8
	9.86	16α-Hydroxy-17a-oxa-d-homo-5α-androstan-3,17-dione (**13**)	–	0.5	5	8
	7.63	16α,17α-Epoxy-progesterone (**14**)^b^	–	–	–	–
	6.96	16α-Hydroxyandrostenedione (**15**)^b^	–	–	–	–
16α-Methoxy-pregnenolone (**16**)	6.23	16α-Methoxy-pregnenolone (**16**)	33	8	1	–
	7.65	16α-Methoxy-progesterone (**17**)	66	86	87	79
	8.13	(20*R*)-20-Hydroxy-16α-methoxy-pregn-4-en-3-one (1**8**)	1	4	12	19

^a^Determined by GC analysis of the crude chloroform extracts

^b^Identified in GC and TLC on the basis of standards

**Fig. 6:**
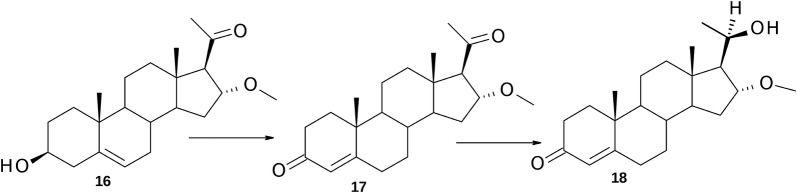
Metabolites isolated following transformation of 16α-methoxy-pregnonolone (**16**) by *P. lanosocoeruleum*

Incubation of 16α-methoxy-pregnenolone (**16**) resulted in a significantly different pattern of metabolism in comparison to other tested C_21_-20-ketones, notably being devoid of side chain degradation (Fig. 6). During the initial period of incubation of **16** the only identifiable metabolite was product of oxidation in A-ring—16α-methoxy-progesterone (**17**). The mixture after 12 h of reaction contained 66% of **17** and it was the highest content of 3-oxo-4-ene metabolite of tested 3β-hydroxy-5-ene steroids. Some amount of this derivative in the next hours was reduced to (20*R*)-20-hydroxy-16α-methoxy-pregn-4-en-3-one (**18**), whose content at the end of the transformation reached only 19%. The stereochemical reduction at C-20 of pregnanes is common to a wide range of fungi [3] and often precedes the reaction of side-chain cleavage to form androstane derivatives. Because the resulting (20*R*)-20-hydroxy-16α-methoxy-pregn-4-en-3-one (**18**) did not undergo further transformation, it can be assumed that in the presence of 16α-methoxy substituent the 20-hydroxy group could not be re-oxidized to the corresponding C-20 ketone and finally BVMO could not be activated. In this case it was probable that intramolecular hydrogen bonds may be inhibiting substrate binding to the oxidase. However, a physical ball-and-stick model demonstrated possibility of only a weak hydrogen interaction between the C-20 hydroxyl group and 16α-methoxy group (more than 3.2 Å), and this reason should be discarded. It is worth noting that we were unable to isolate the corresponding 20-alcohols from other tested steroids. This may indicate that progesterone (**1**) and pregnenolones **5**, **8** and **9** do not activate reductase or that their molecular structure is precluding binding to this enzyme. On the other hand, the mentioned substrates were metabolized at a faster rate than **16** by side-chain degrading enzymes (Tables 1, 2) and therefore accumulation of 20-dihydro compounds was prevented.

During the experiments carried out to elucidate the pattern of metabolic processing of steroidal compounds by *P. lanosocoeruleum* KCH3012, we unexpectedly noticed that there may result activation of silent biosynthetic pathways for production of polyketide metabolites. From fermentation medium after 4 days transformation of pregnenolone (**5**) we isolated in large yield a 12-membered macrolactone—(*S*)-curvularin (**19**). Curvularin and its structural relatives are produced by a number of phytopatogenic fungi from such genera as *Curvularia*, *Alternaria* and *Penicillium* [37], but this is the first report of the occurrence of curvularin in the species *P. lanosocoeruleum*. It is apparent that further investigation is required and we hope to provide detailed information of this finding in future reports.

## Conclusions

This work and previous studies with *P. lanosocoeruleum* KCH 3012 clearly demonstrated that this microorganism has potent multi-functional catalytic properties towards androstane- and pregnane-type steroids. It contains (in similarity to *P. lilacinum* AM111 [18], *P. camembertii* AM83 [19], *P. simplicissimum* WY134-2 [22]) an endogenous lactonization pathway which can transform progesterone to testololactone with high yield. In this microorganism a steroidal 3β-HSD was active, and as a result the same metabolite (although with lower yield) was obtained from pregnenolone. This is in contrast to the result with *P. citreo*-*viride* ACCC 0402 where pregnenolone was not transformed [17] or with *P. lilacinum* AM111 which oxidized pregnenolone to 3β-hydroxy-17a-oxa-d-homo-androst-5-en-17-one—a d-lactone with conserved 5-en-3β-OH moiety [18]. The tested microorganism was able to attack side chains of steroids bearing substituents at C-16. Moreover, studies have highlighted that a slight modification of the D-ring of the substrate may induce and control metabolic fate either into the lactonization or reductive and oxidative pathways. The presence of double bond at C-16 appears to stimulate the rate of metabolism in the D-ring, and as consequence, the transformation of 16-dehydropregnenolone yields 3β-hydroxy-5-ene D-lactone. Importantly, our studies have demonstrated for the first time that incubation of 16α,17α-epoxy-pregnenolone with a fungus strain belonging to the genus *Penicillium* resulted in a mixture of 16α-hydroxy lactones. Possibility of epoxide opening by enzymes from this fungus affords a unique opportunity for generation of novel bioactive steroidal compounds. Furthermore, we showed that no steroidal lactone was formed after transformation of 16α-methoxy-pregnenolone. This would suggest that the presence of the 16α-methoxy group prevents the side chain cleavage in C_21_ steroids.

## Methods

### Materials

Progesterone (**1**), pregnenolone (**5**), 16α,17α-epoxy-pregnenolone (**9**), 16α,17α-epoxy-progesterone (**14**), and 16-dehydropregnenolone acetate (**20**) were purchased from Sigma-Aldrich Chemical Co. Testosterone (**2**), androstenedione (**3**), DHEA (dehydroepiandrosterone) (**6**) were purchased from Steraloids Inc. 16α-Hydroxyandrostenedione (**15**) was obtained in our previous work by transformation of testosterone using *Aspergillus niger* KCH910 [38]. The last four of the aforementioned compounds and **14** were used as analytical standards for the time course experiments. 16-Dehydro-pregnenolone (**8**) and 16α-methoxy-pregnenolone (**16**) (in a ratio of 3:7) were prepared from the 16-dehydropregnenolone acetate (**20**) by its saponification with potassium hydroxide in methanol [39]. The resulting mixture was chromatographed on a column of silica with cyclohexane/chloroform/diethyl ether (1:0.75:1 v/v/v) as eluent. The products were found to be in excess of 98.5 and 97.2% purity following GC analysis.

#### 16-Dehydro-pregnenolone (**8**)

^1^H NMR (300 MHz, CDCl_3_) δ_H_: 0.90 (3H, s, 18-H), 1.03 (3H, s, 19-H), 2.25 (3H. s, 21-H), 3.52 (1H, m, 3α-H), 5.35 (1H, d, *J *= 4.3 Hz, 6-H), 6.70 (1H, s, 16-H). ^13^C NMR (75 MHz, CDCl_3_): 15.7 (C-18), 19.3 (C-19), 20.7 (C-11), 27.1 (C-21), 30.2 (C-8), 31.5 (C-7), 31.6 (C-2), 32.2 (C-15), 34.6 (C-12), 36.7 (C-10), 37.1 (C-1), 42.2 (C-4); 46.1 (C-13); 50.4 (C-9), 56.4 (C-14), 71.7 (C-3), 121.1 (C-6), 141.3 (C-5), 144.5 (C-16), 155.6 (C-17), 196.9 (C-20). NMR data was found to be in good agreement with that described by Szendi [40].

#### 16α-Methoxy-pregnenolone (**16**)

^1^H NMR (300 MHz, CDCl_3_) δ_H_: 0.62 (3H. s, 18-H), 0.99 (3H, s, 19-H), 2.17 (3H. s, 21-H), 2.53 (1H, d, *J *= 3.0 Hz, 17α-H), 3.20 (1H, s, 16α-OCH_3_), 3.50–3.54 (1H, m, 3α-H), 4.33–4.35 (1H, m, 16β-H), 5.34 (1H, d, *J *= 2.7 Hz, 6-H). ^13^C NMR (75 MHz, CDCl_3_): 14.4 (C-18), 19.3 (C-19), 20.7 (C-11), 31.4 (C-8), 31.5 (C-2, C-15), 31.7 (C-21), 31.9 (C-7), 36.5 (C-10), 37.1 (C-1), 38.8 (C-12), 42.2 (C-4), 44.4 (C-13), 49.8 (C-9), 54.4 (C-14), 57.1 (C-22), 71.6 (C-3, C-17), 81.4 (C-16), 121.2 (C-6), 140.7 (C-5), 208.1 (C-20). NMR data was found to be identical with that given by Wölfling [41].

The fungal strain *Penicillium lanosocoeruleum* KCH 3012 used in this study was taken from the collection of the Department of Chemistry, Wrocław University of Environmental and Life Sciences (Wrocław, Poland). The fungus was maintained on Sabouraud 4% dextrose-agar slopes at 4 °C and freshly subcultured before use in the transformation experiments.

### General conditions of cultivation and transformation

General experimental and fermentation details were described previously [18]. Each substrate was added to a 72-h-old culture of the microorganism as an acetone solution, in concentration of 0.16 mmol/100 mL of medium, and incubated for 3–4 days (until the contents of the substrate in the reaction mixture reached stationary level) at 25 °C in a rotary shaker (180 rpm). Each experiment was performed with three replications.

### Isolation and identification of the products

The products of biotransformation were extracted three times with ethyl acetate. The organic extracts were dried over anhydrous magnesium sulfate, concentrated *in vacuo* and analyzed by TLC and GC. Transformation products were separated by column chromatography on silica gel with ethyl acetate:methylene chloride:acetone (3:1:1 v:v:v) for progesterone (**1**) and pregnonolone (**5**), hexane:ethyl acetate:isopropyl alcohol (2:0.5:0.5 v:v:v) for 16-dehydro-pregnenolone (**8**), ethyl acetate:diethyl ether (2:1 v:v) for 16α,17α-epoxypregnenolone (9), or hexane:ethyl acetate:isopropyl alcohol (1:0.15:0.1 v:v:v) for 16α-methoxy-pregnenolone (**16**) as eluents. TLC was carried out with Kieselgel 60 F_254_ plates (Merck, Darmstadt, Germany) using the same eluents. In order to develop the image, the plates were sprayed with solution of methanol in concentrated sulfuric acid (1:1) and heated to 120 °C for 3 min. GC analysis was performed using Hewlett Packard 5890A Series II GC instrument (FID, carrier gas H_2_ at flow rate of 2 mL min^−1^) with DB-5MS column cross-linked phenyl-methylsiloxane, 30 m × 0.32 mm × 0.25 μm film thickness. The following program was used in the GC analysis: 220 °C/1 min, gradient 4 °C/min to 280° and 30 °C/min to 300°/2 min (for **1**, **5** and **8**) or 230 °C/1 min, gradient 4 °C/min to 280° and 30 °C/min to 300°/2 min (for **9** and **16**); injector and detector temperatures were 300 °C. The NMR spectra were measured in CDCl_3_ or CD_3_OD and recorded on a DRX 300 MHz Bruker Avance spectrometer. Characteristic ^1^H- and ^13^C-NMR shift values in comparison to the starting compounds were used to determine structures of metabolites, in combination with DEPT analysis to identify the nature of the carbon atoms. Optical rotation measurements were carried out on Autopol IV automatic polarimeter (Rudolph).

### Time course experiments

For studying the time-dependent progress of the bioconversion and to determine the metabolic pathways of substrates, 5-mL samples of the broth were taken out at regular intervals from the reaction flask, extracted with ethyl acetate and analyzed by comparison of the GC and TLC data with those of authentic samples. Conditions of the reaction were identical to those in the main biotransformation experiments.

## Additional file

**Additional file 1.** ^1^H, ^13^C, DEPT and NOESY NMR spectra of the biotransformation products.
