# Supplementary material for: Metabolic fate of pregnene-based steroids in the lactonization pathway of multifunctional strain Penicillium lanosocoeruleum
Source: Microb Cell Fact. 2018 Jun 26;17:100. doi: 10.1186/s12934-018-0948-1 (PMC6019235; doi:10.1186/s12934-018-0948-1)
Supplement: Supplementary file 1 — Additional file 1. 1H, 13C, DEPT and NOESY NMR spectra of the biotransformation products. [file 12934_2018_948_MOESM1_ESM.pdf]

Additional file 1

Metabolic fate of pregnene-based steroids in the lactonization pathway of multifunctional strain *Penicillium lanosocoeruleum*

Alina Świzdor, Anna Panek, Paulina Ostrowska*

Department of Chemistry, Wrocław University of Environmental and Life Sciences, Norwida 25, 50-375 Wrocław, Poland

Contents:

S1.^{1}H NMR spectrum of testololactone (**4**) 4

S2.^{13}C NMR spectrum of testololactone (**4**) 4

S3.^{1}H NMR spectrum of 3β-hydroxy-17a-oxa-D-homo-androst-5-en-17-one (**7**). 5

S4.^{13}C NMR spectrum of 3β-hydroxy-17a-oxa-D-homo-androst-5-en-17-one (**7**) 5

S5.^{1}H NMR spectrum of 16-dehydro-pregnenolone (**8**) 6

S6.^{13}C NMR spectrum of 16-dehydro-pregnenolone (**8**) 6

S7.^{1}H NMR spectrum of 3β,16α-dihydroxy-17a-oxa-D-homo-androst-5-en-17-one (**10**) 7

S8.^{13}C NMR spectrum of 3β,16α-dihydroxy-17a-oxa-D-homo-androst-5-en-17-one (**10**) 7

S9. DEPT spectrum of 3β,16α-dihydroxy-17a-oxa-D-homo-androst-5-en-17-one (**10**) 8

S10. NOESY spectrum of 3β,16α-dihydroxy-17a-oxa-D-homo-androst-5-en-17-one (**10**) 8

S11.^{1}H NMR spectrum of 16α-hydroxy-17a-oxa-D-homo-androst-4-en-3,17-dione (**11**) 9

S12.^{13}C NMR spectrum of 16α-hydroxy-17a-oxa-D-homo-androst-4-en-3,17-dione (**11**) 9

S13. DEPT spectrum of 16α-hydroxy-17a-oxa-D-homo-androst-4-en-3,17-dione (**11**) 10

S14. NOESY spectrum of 16α-hydroxy-17a-oxa-D-homo-androst-4-en-3,17-dione (**11**) 10

S15.^{1}H NMR spectrum of 3β,16α-dihydroxy-17a-oxa-D-homo-5α-androstan-17-one (**12**) ... 11

S16.^{13}C NMR spectrum of 3β,16α-dihydroxy-17a-oxa-D-homo-5α-androstan-17-one (**12**) .. 11

S17. DEPT spectrum of 3β,16α-dihydroxy-17a-oxa-D-homo-5α-androstan-17-one (**12**) 12

S18. NOESY spectrum of 3β,16α-dihydroxy-17a-oxa-D-homo-5α-androstan-17-one (**12**) ... 12

S19.^{1}H NMR spectrum of 16α-hydroxy-17a-oxa-D-homo-5α-androstan-3,17-dione (**13**) 13

S20.^{13}C NMR spectrum of 16α-hydroxy-17a-oxa-D-homo-5α-androstan-3,17-dione (**13**) 13

S21. DEPT spectrum of 16α-hydroxy-17a-oxa-D-homo-5α-androstan-3,17-dione (**13**).......... 14

S22. NOESY spectrum of 16α-hydroxy-17a-oxa-D-homo-5α-androstan-3,17-dione (**13**) 14

S23.^{1}H NMR spectrum of 16α-methoxy-pregnenolone (**16**) 15

S24.^{13}C NMR spectrum of 16α-methoxy- pregnenolone (**16**) 15

S25.^{1}H NMR spectrum of 16α-methoxy-progesterone (**17**) 16

S26.^{13}C NMR spectrum of 16α-methoxy- progesterone (**17**) 16

S27.DEPT spectrum of 16α-methoxy-progesterone (**17**).. 17

S28.^{1}H NMR spectrum of (20*R*)-20-hydroxy-16α-methoxy-pregn-4-en-3-one (**18**) 17

S29.^{13}C NMR spectrum of (20*R*)-20-hydroxy-16α-methoxy-pregn-4-en-3-one (**18**) 18

S30.DEPT spectrum of (20*R*)-20-hydroxy-16α-methoxy-pregn-4-en-3-one (**18**).................. 18

S31.HSQC spectrum of (20*R*)-20-hydroxy-16α-methoxy-pregn-4-en-3-one (**18**) 19

S32.NOESY spectrum of (20*R*)-20-hydroxy-16α-methoxy-pregn-4-en-3-one (**18**) 19

S33.^{1}H NMR spectrum of (*S*)-curvularin (**19**) 20

S34.^{13}C NMR spectrum of (S)-curvularin (**19**) 20

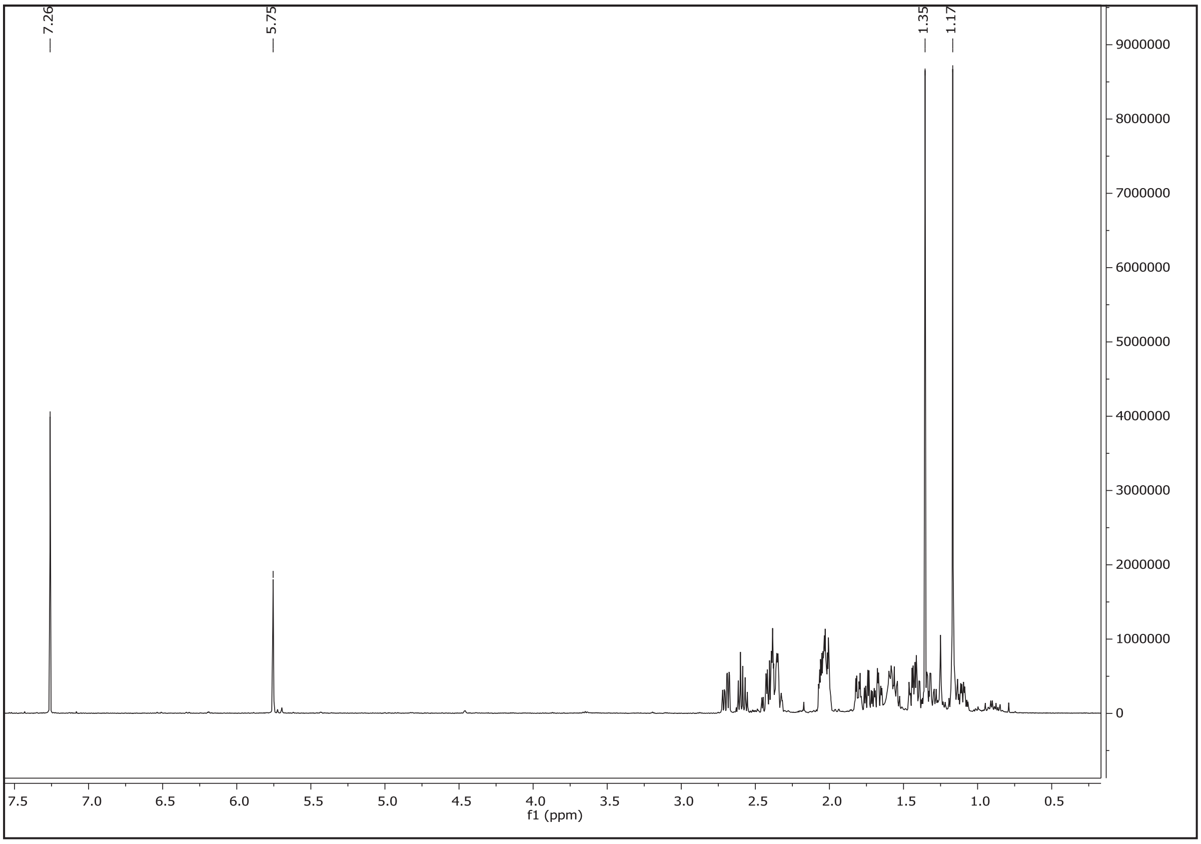

S1. ^{1}H NMR spectrum of testololactone (**4**)

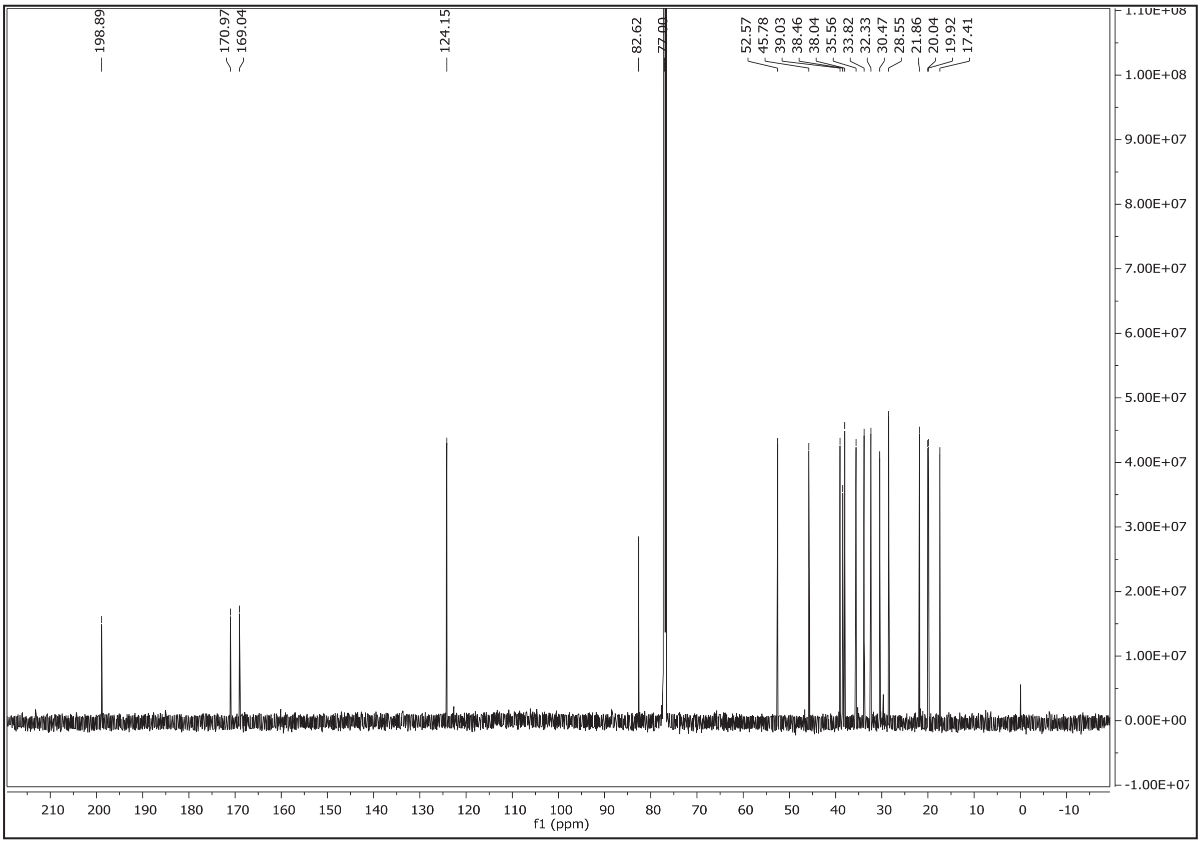

S2. ^{13}C NMR spectrum of testololactone (**4**)

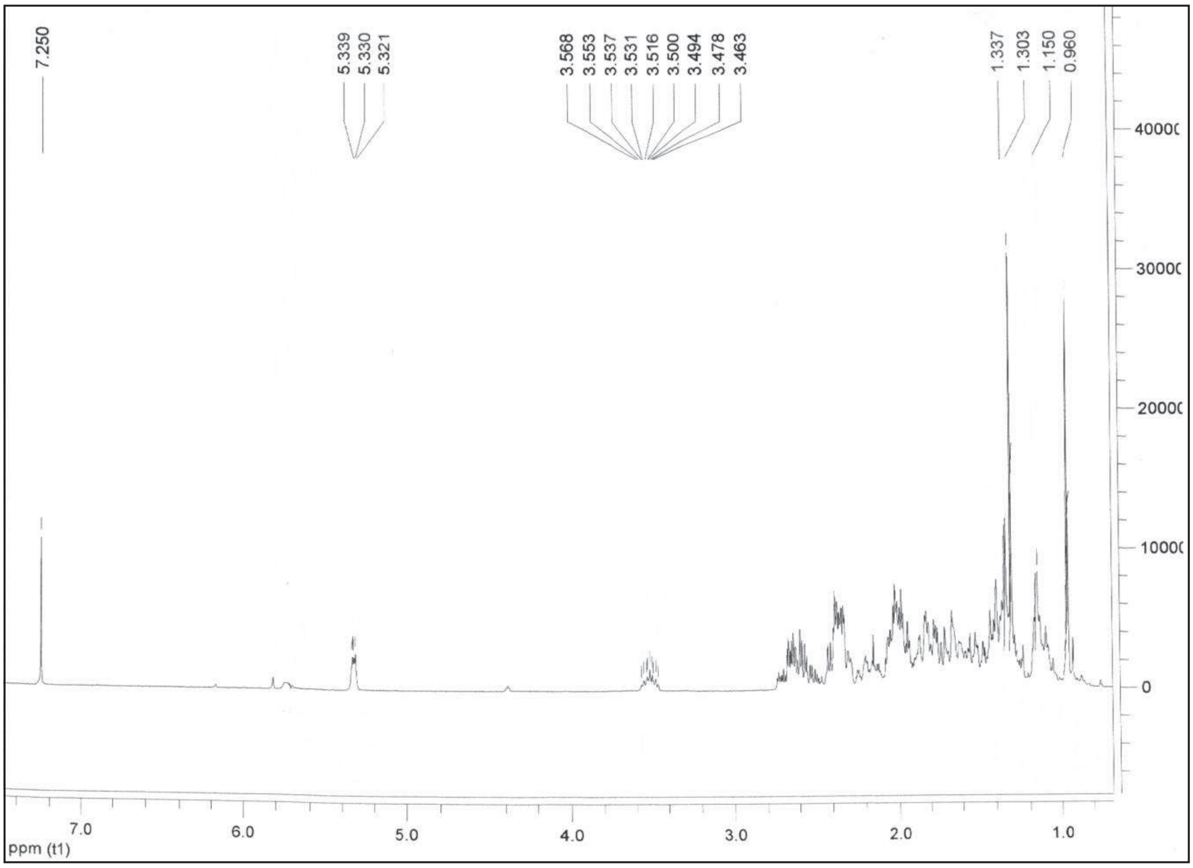

S3. ^{1}H NMR spectrum of 3β-hydroxy-17a-oxa-D-homo-androst-5-en-17-one (**7**)

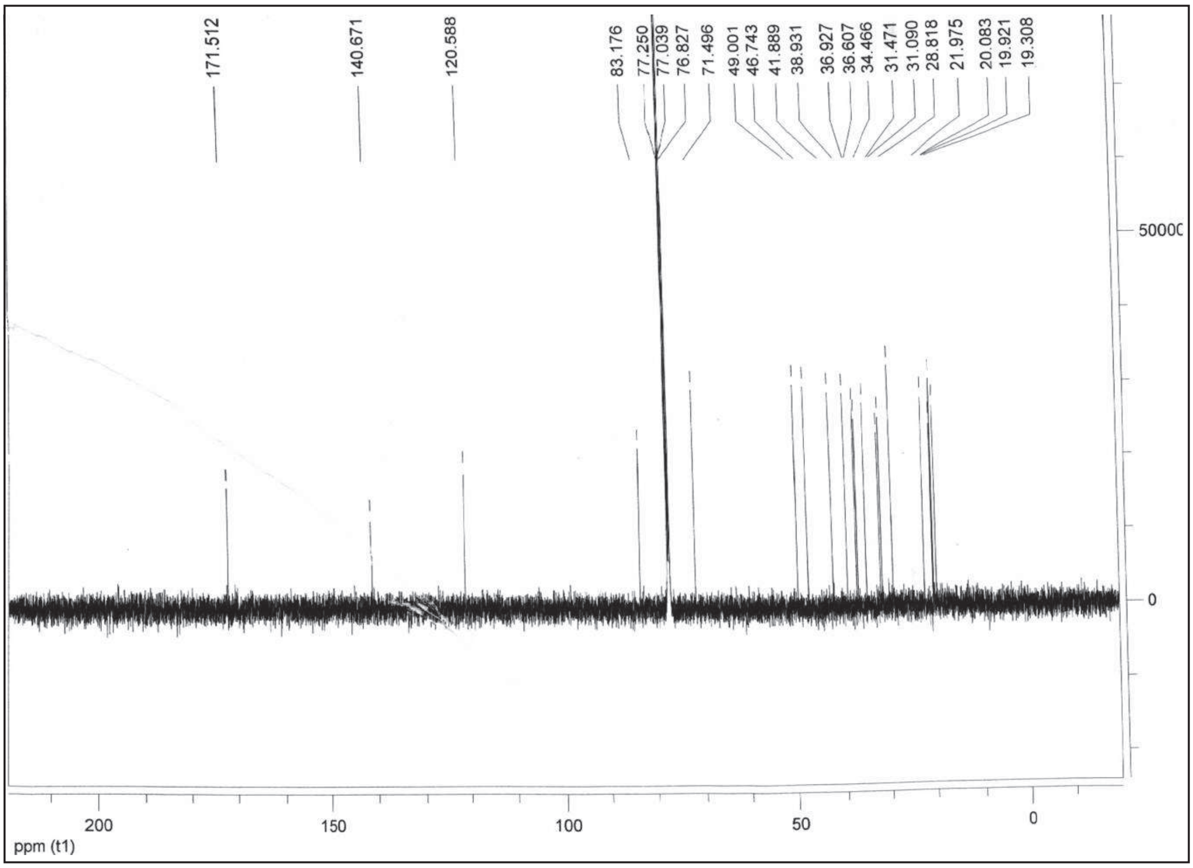

S4. ^{13}C NMR spectrum of 3β-hydroxy-17a-oxa-D-homo-androst-5-en-17-one (**7**)

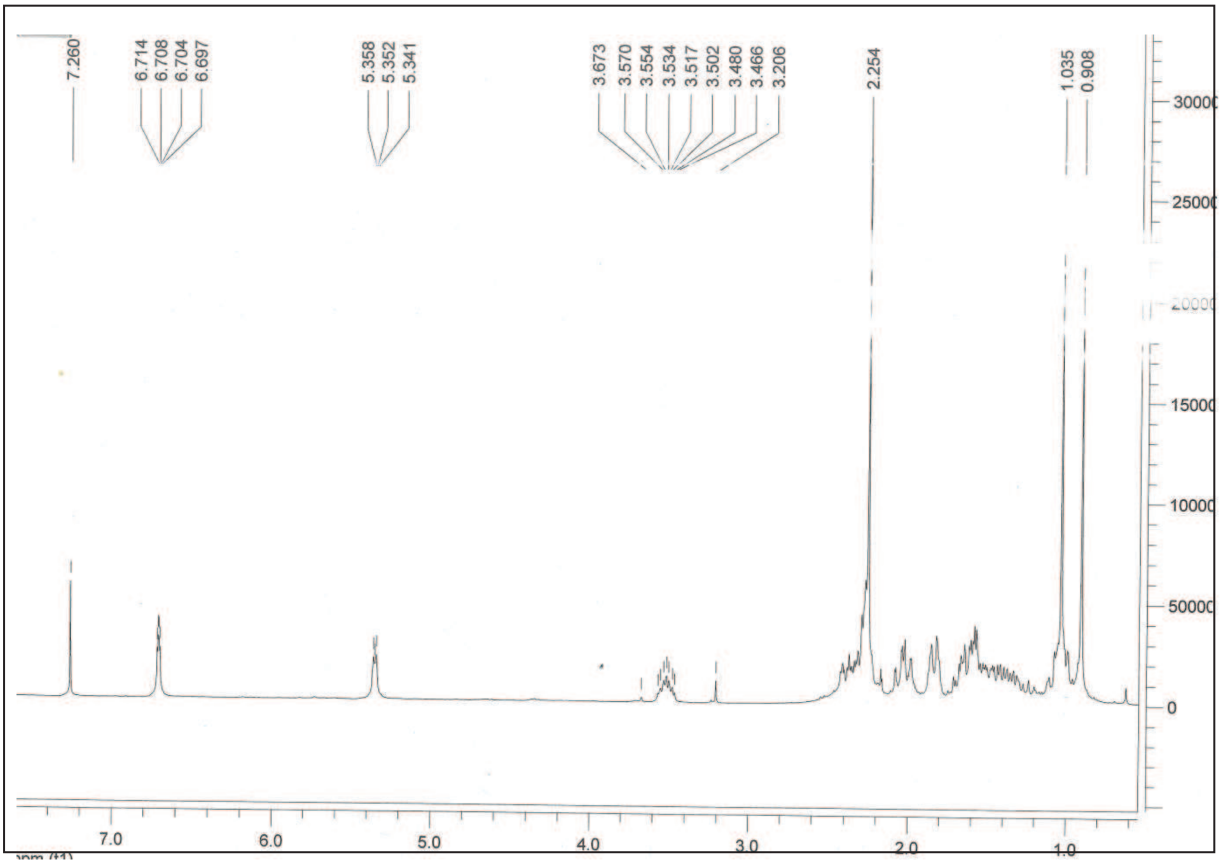

S5. ^{1}H NMR spectrum of 16-dehydro-pregnenolone (**8**).

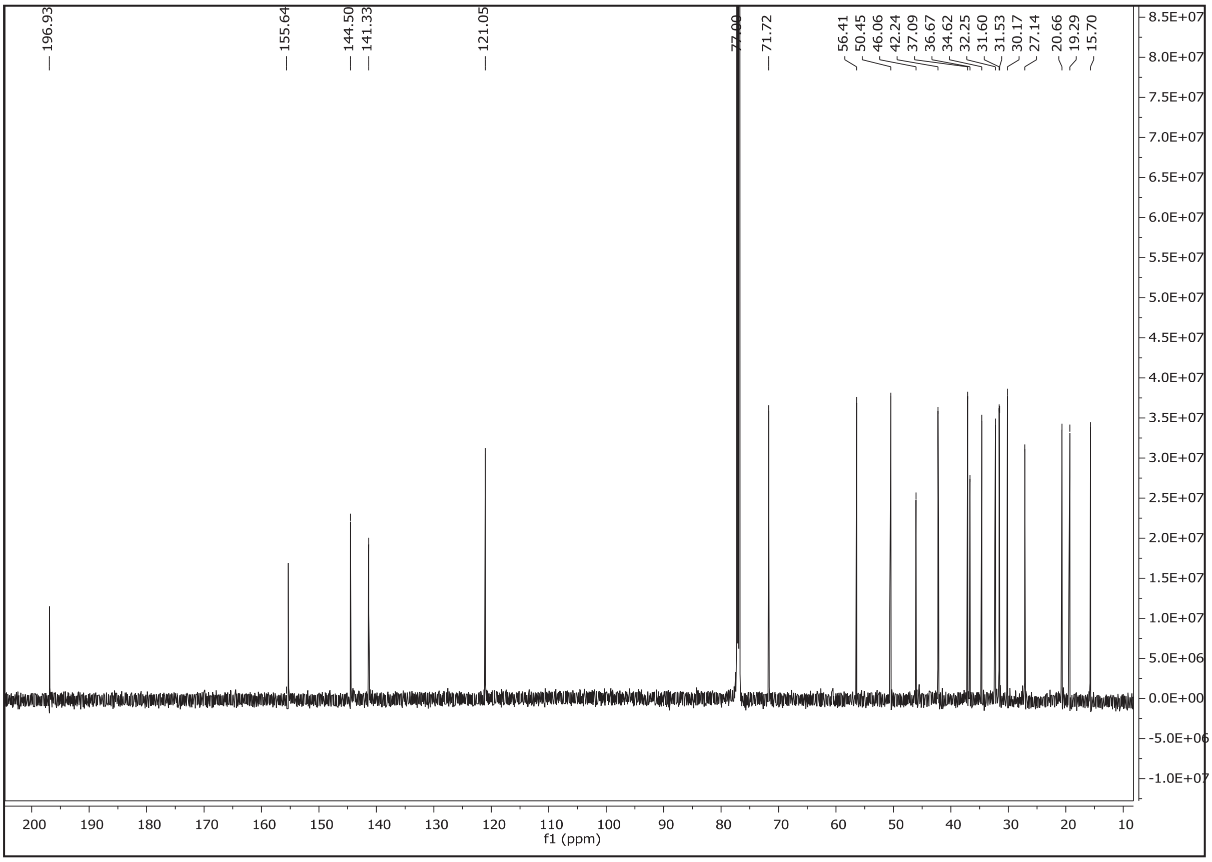

S6. ^{13}C NMR spectrum of 16-dehydro-pregnenolone (**8**).

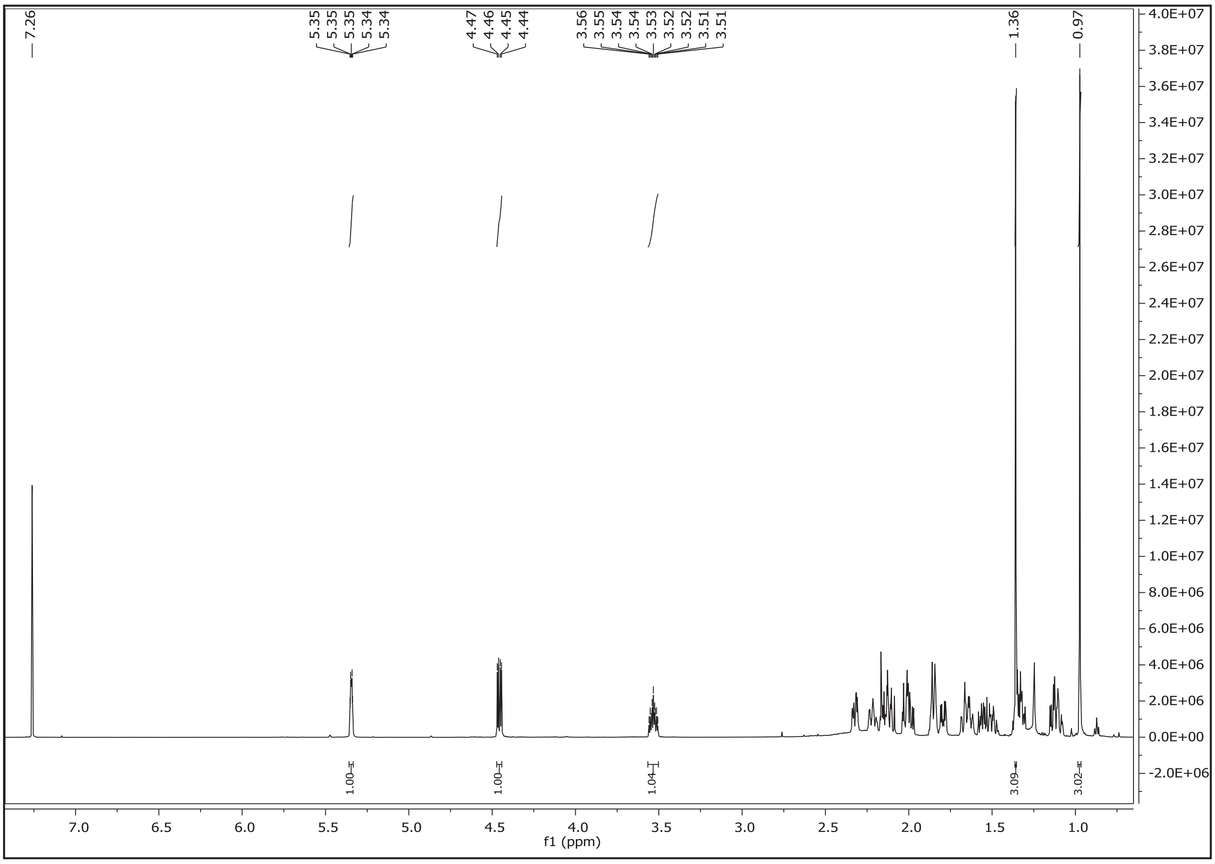

S7. ^{1}H NMR spectrum of 3β,16α-dihydroxy-17a-oxa-D-homo-androst-5-en-17-one (**10**)

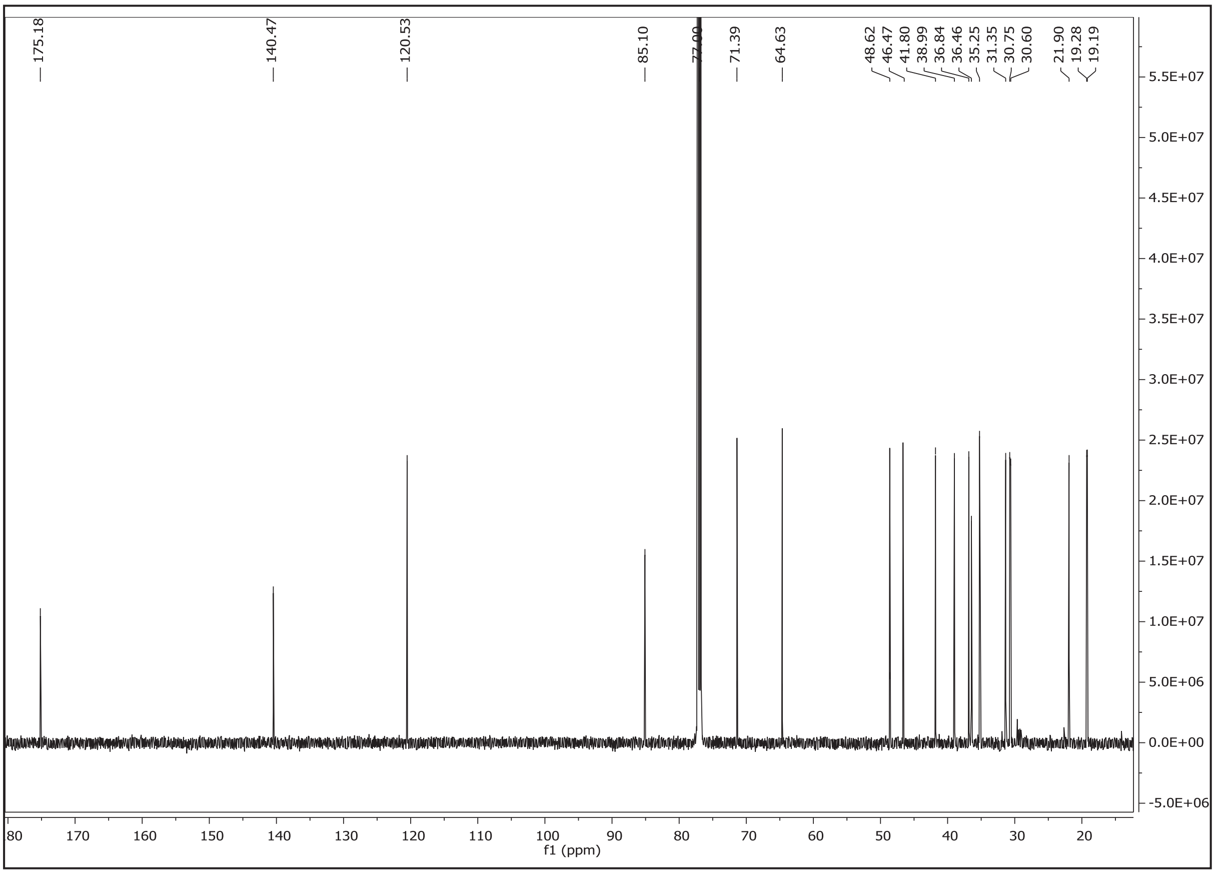

S8. ^{13}C NMR spectrum of 3β,16α-dihydroxy-17a-oxa-D-homo-androst-5-en-17-one (**10**)

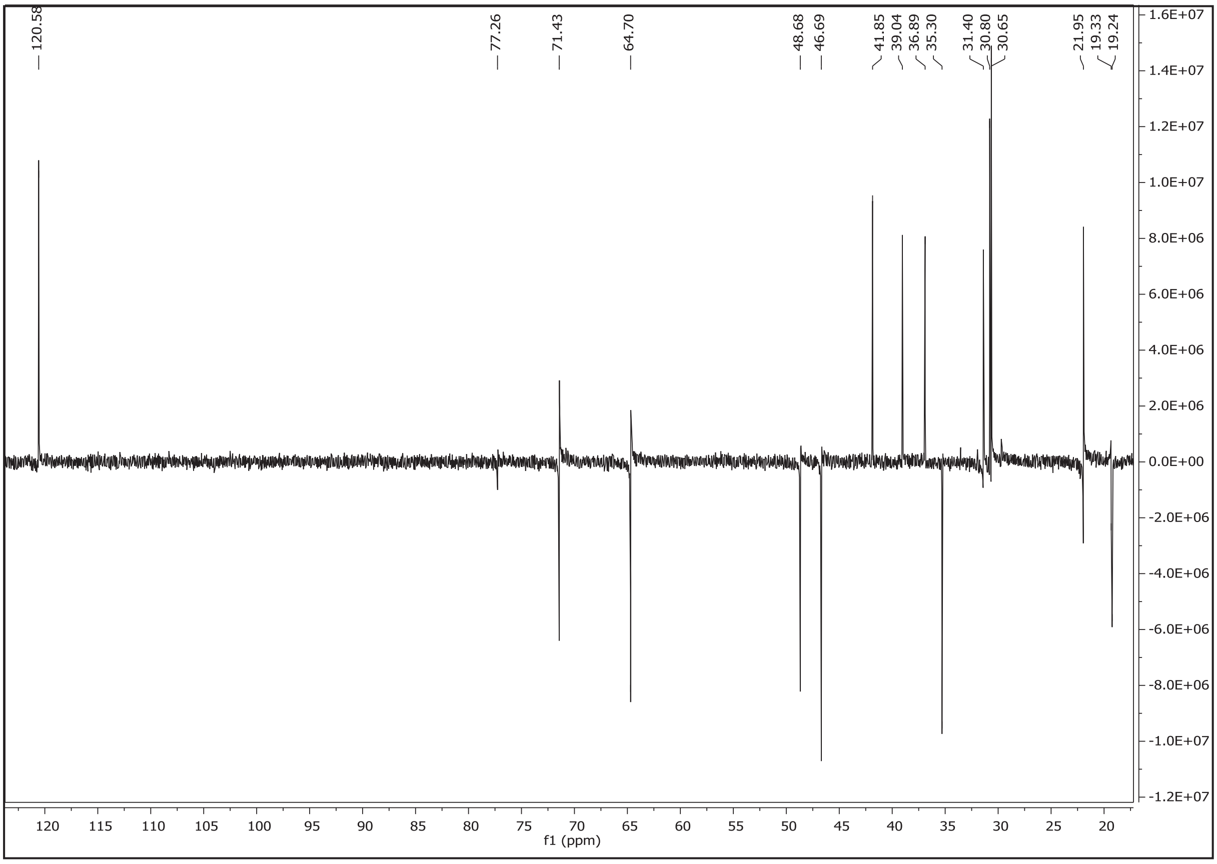

S9. DEPT spectrum of 3β,16α-dihydroxy-17a-oxa-D-homo-androst-5-en-17-one (**10**)

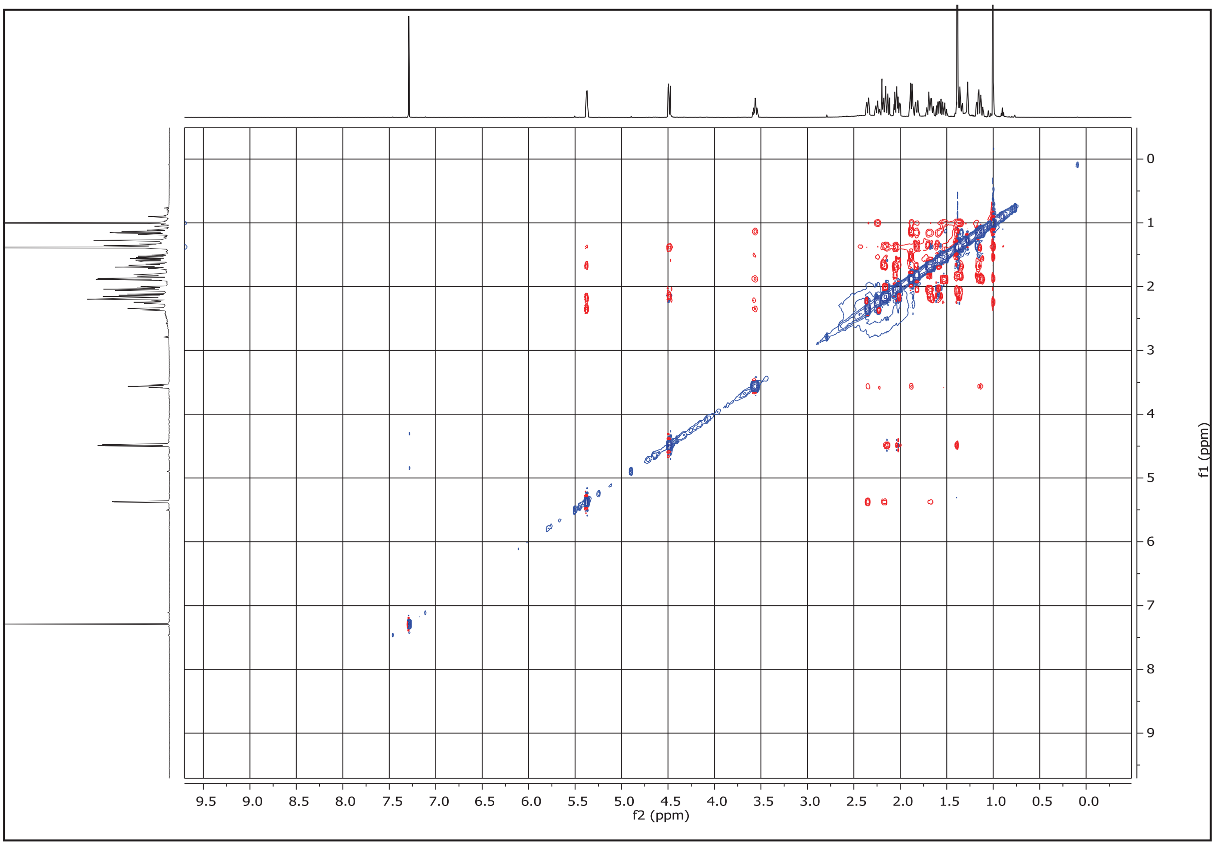

S10. NOESY spectrum of 3β,16α-dihydroxy-17a-oxa-D-homo-androst-5-en-17-one (**10**)

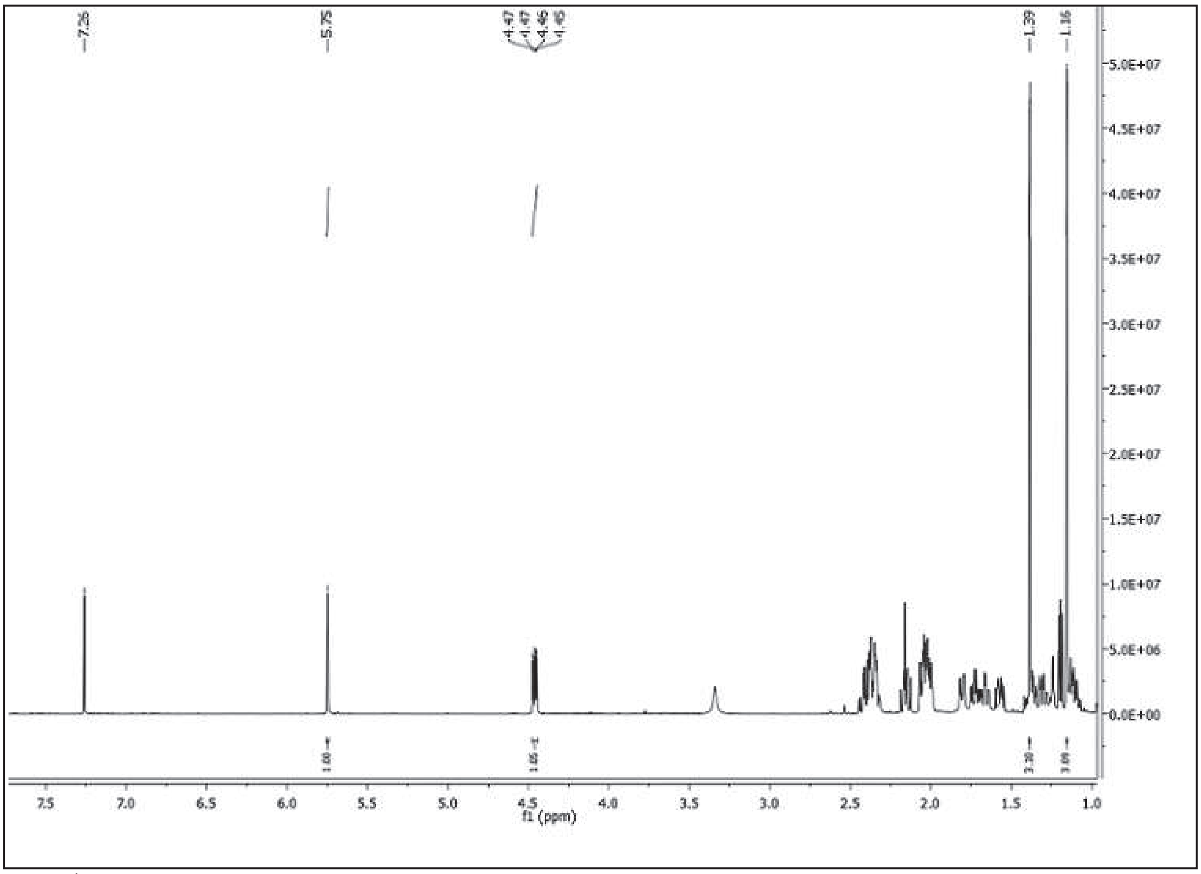

S11. ^{1}H NMR spectrum of 16α-hydroxy-17a-oxa-D-homo-androst-4-en-3,17-dione (**11**)

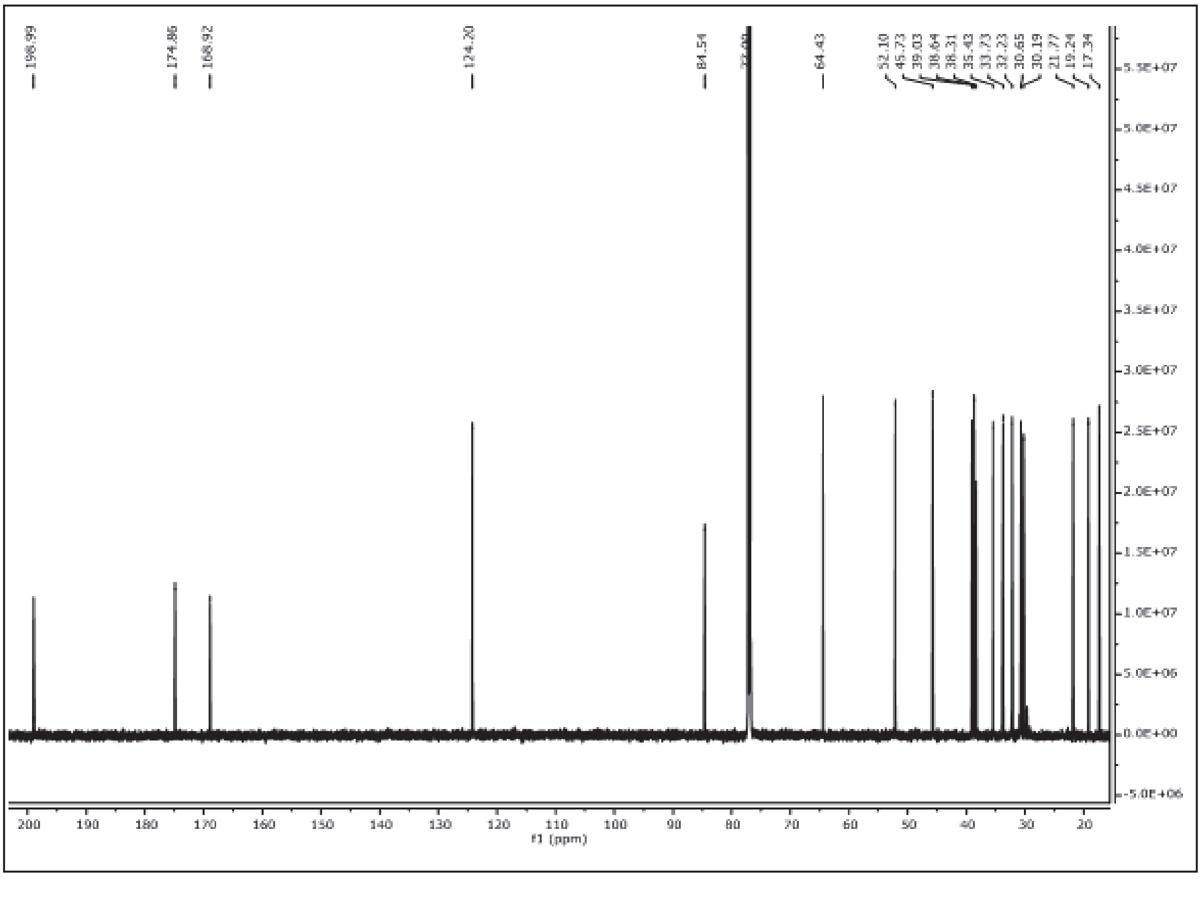

S12. ^{13}C NMR spectrum of 16α-hydroxy-17a-oxa-D-homo-androst-4-en-3,17-dione (**11**)

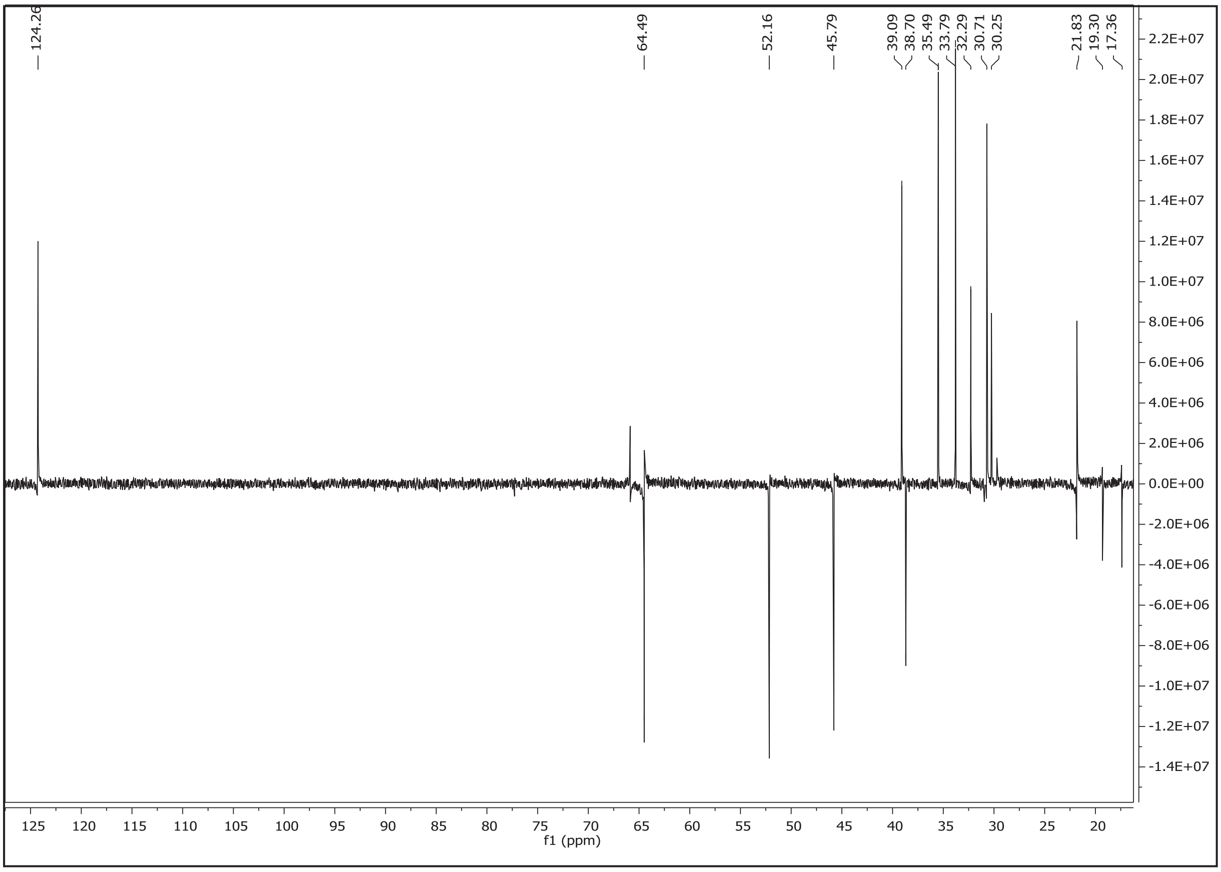

S13. DEPT spectrum of 16α-hydroxy-17a-oxa-D-homo-androst-4-en-3,17-dione (**11**)

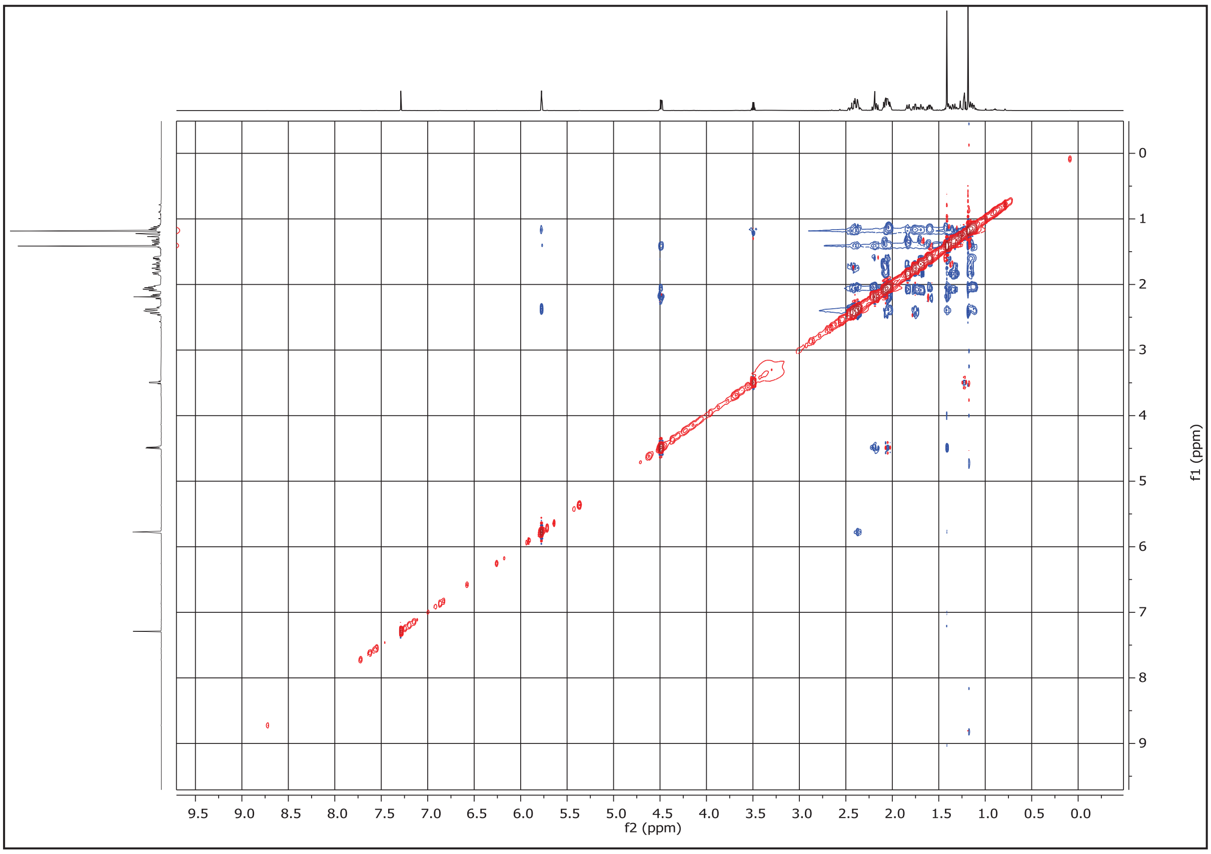

S14. NOESY spectrum of 16α-hydroxy-17a-oxa-D-homo-androst-4-en-3,17-dione (**11**)

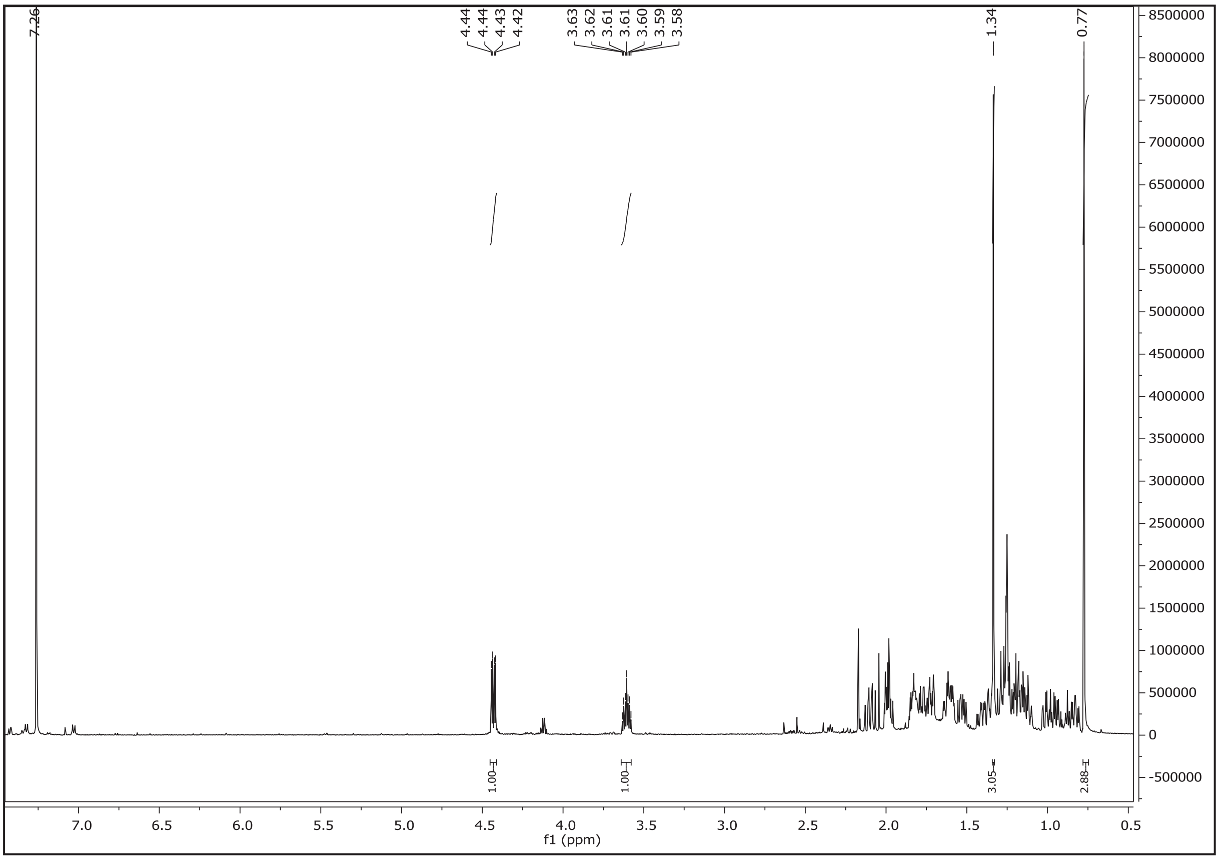

S15. ^{1}H NMR spectrum of 3β,16α-dihydroxy-17a-oxa-D-homo-5α-androstan-17-one (**12**)

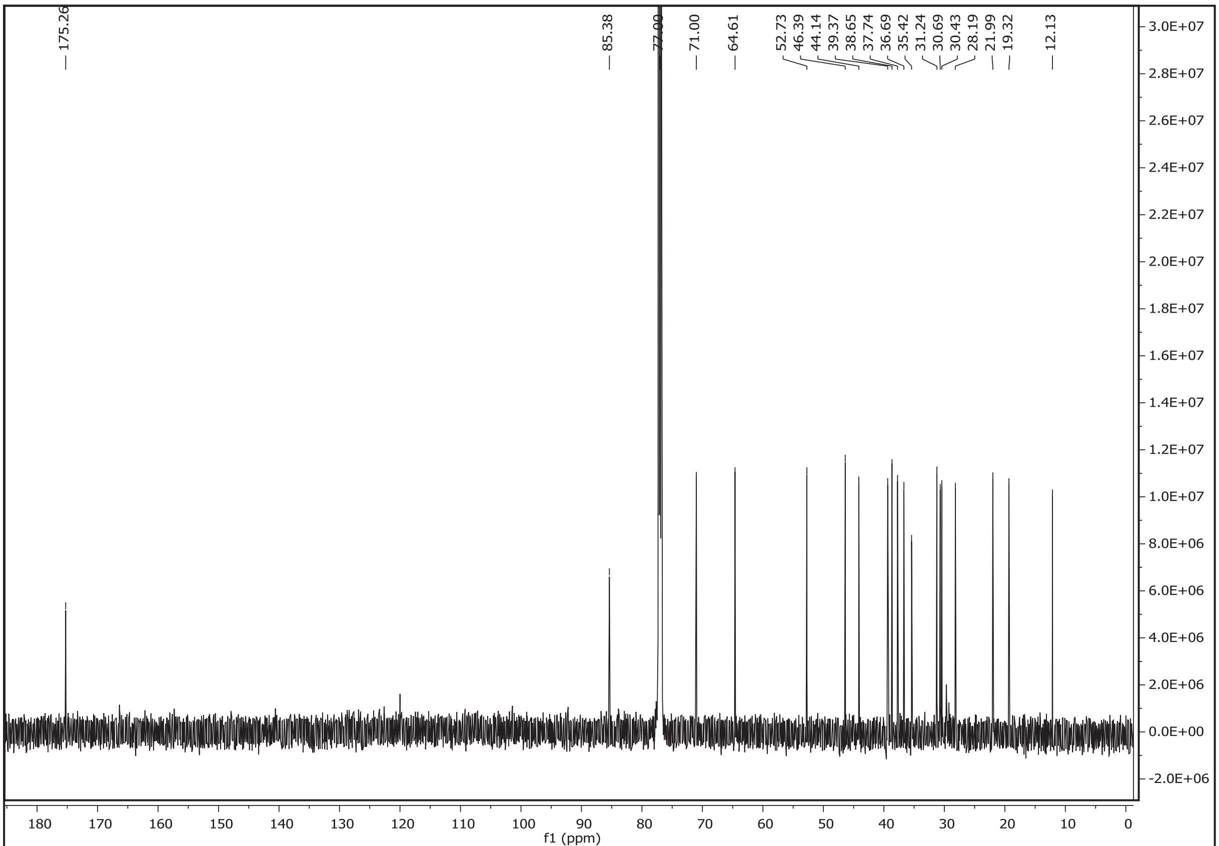

S16. ^{13}C NMR spectrum of 3β,16α-dihydroxy-17a-oxa-D-homo-5α-androstan-17-one (**12**)

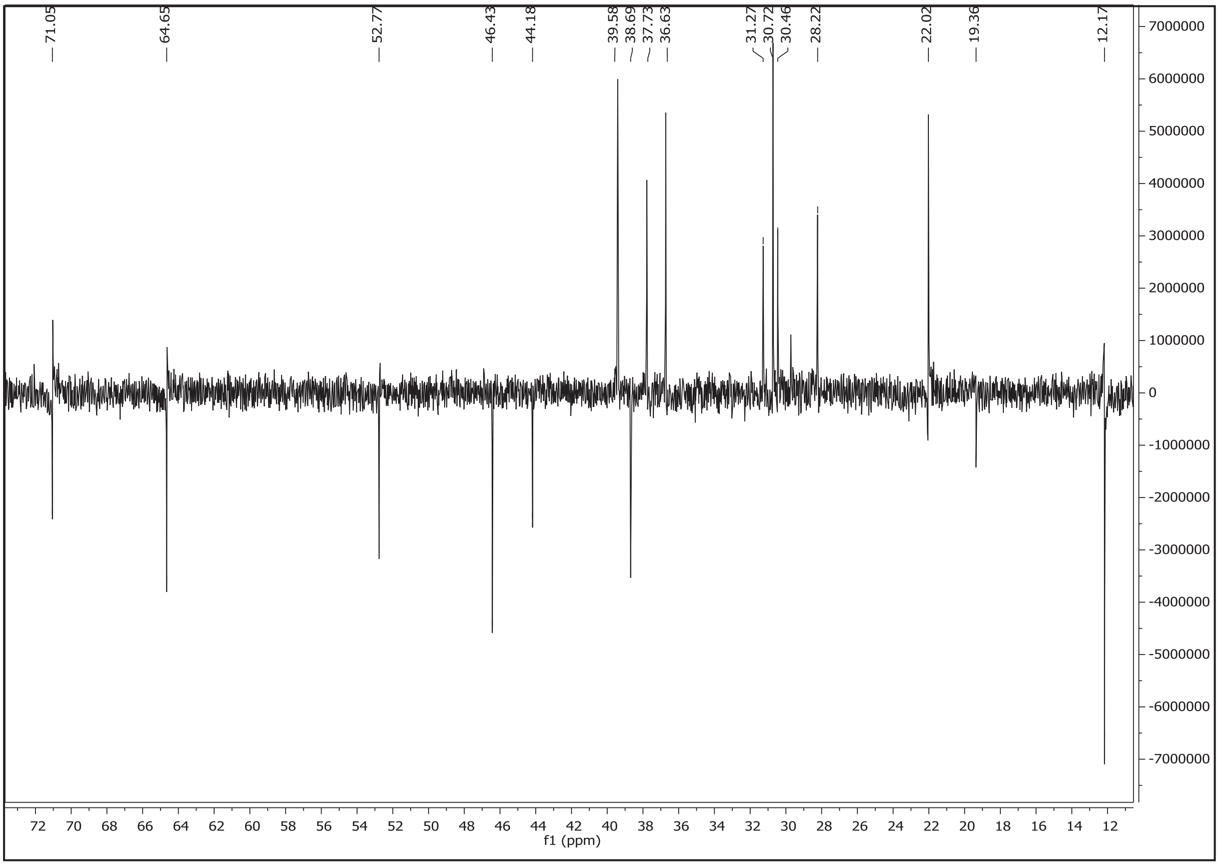

S17. DEPT spectrum of 3β,16α-dihydroxy-17a-oxa-D-homo-5α-androstan-17-one (**12**)

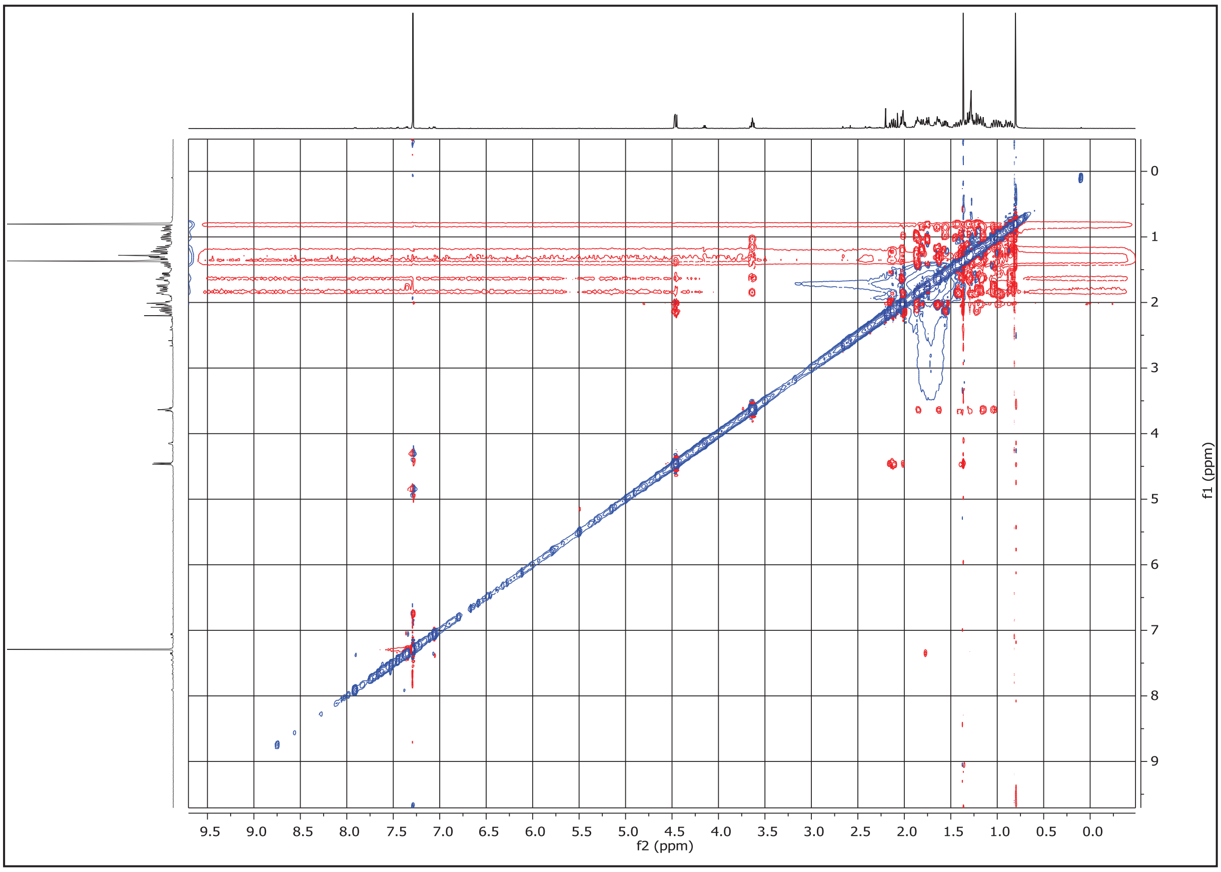

S18. NOESY spectrum of 3β,16α-dihydroxy-17a-oxa-D-homo-5α-androstan-17-one (**12**)

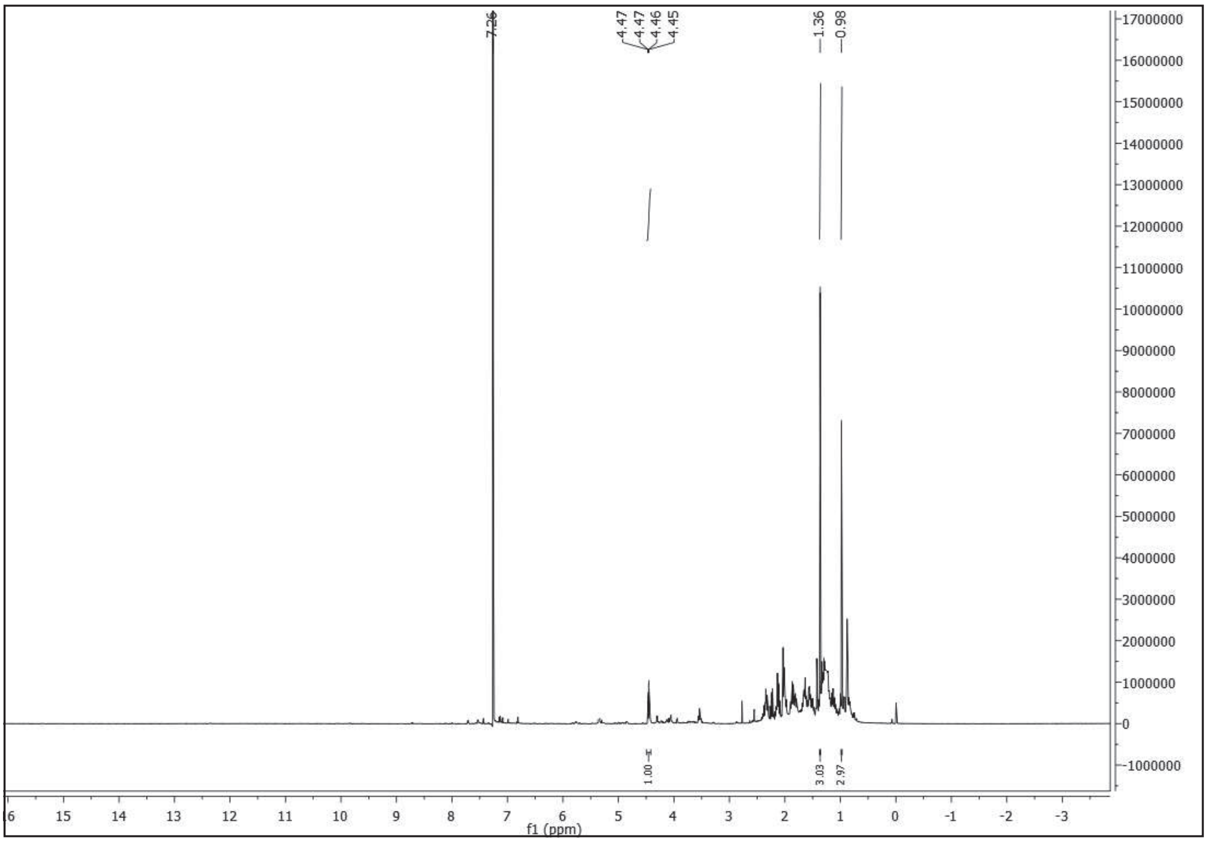

S19. ^{1}H NMR spectrum of 16α-hydroxy-17a-oxa-D-homo-5α-androstan-3,17-dione (**13**)

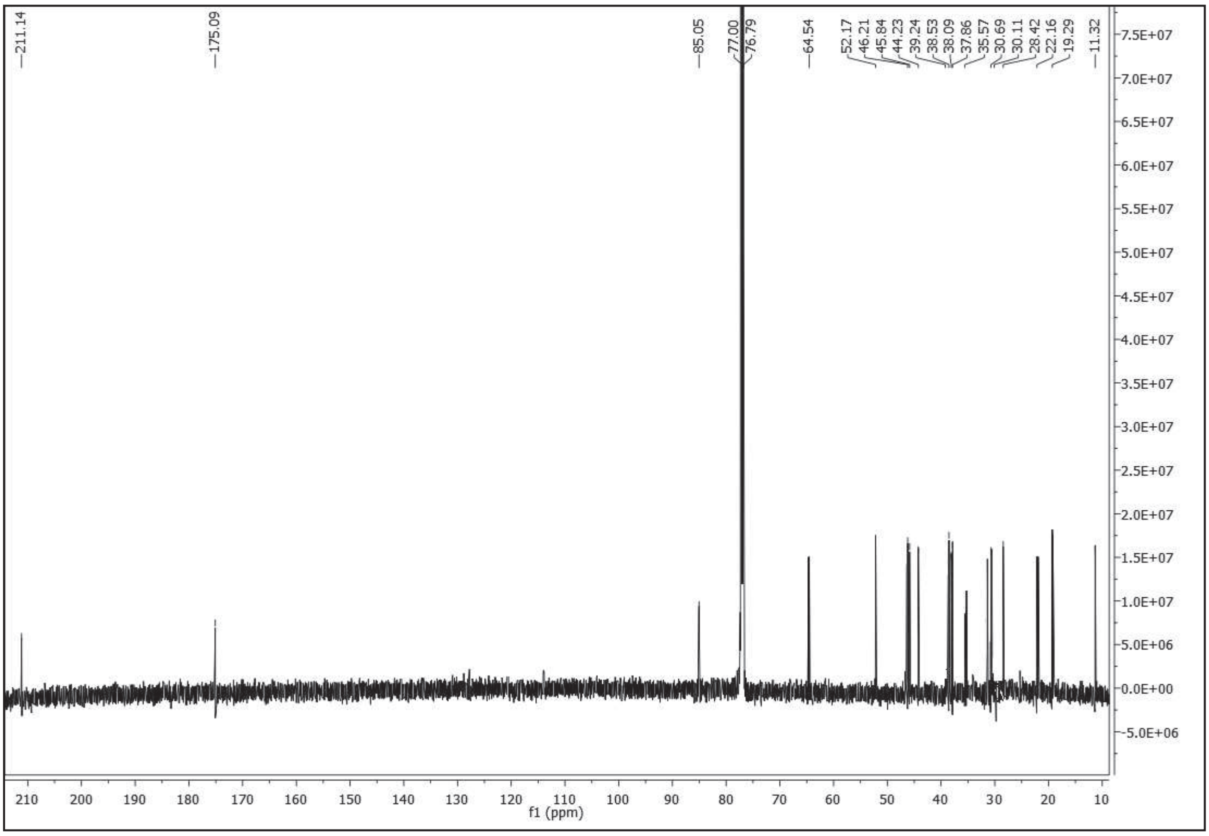

S20. ^{13}C NMR spectrum of 16α-hydroxy-17a-oxa-D-homo-5α-androstan-3,17-dione (**13**)

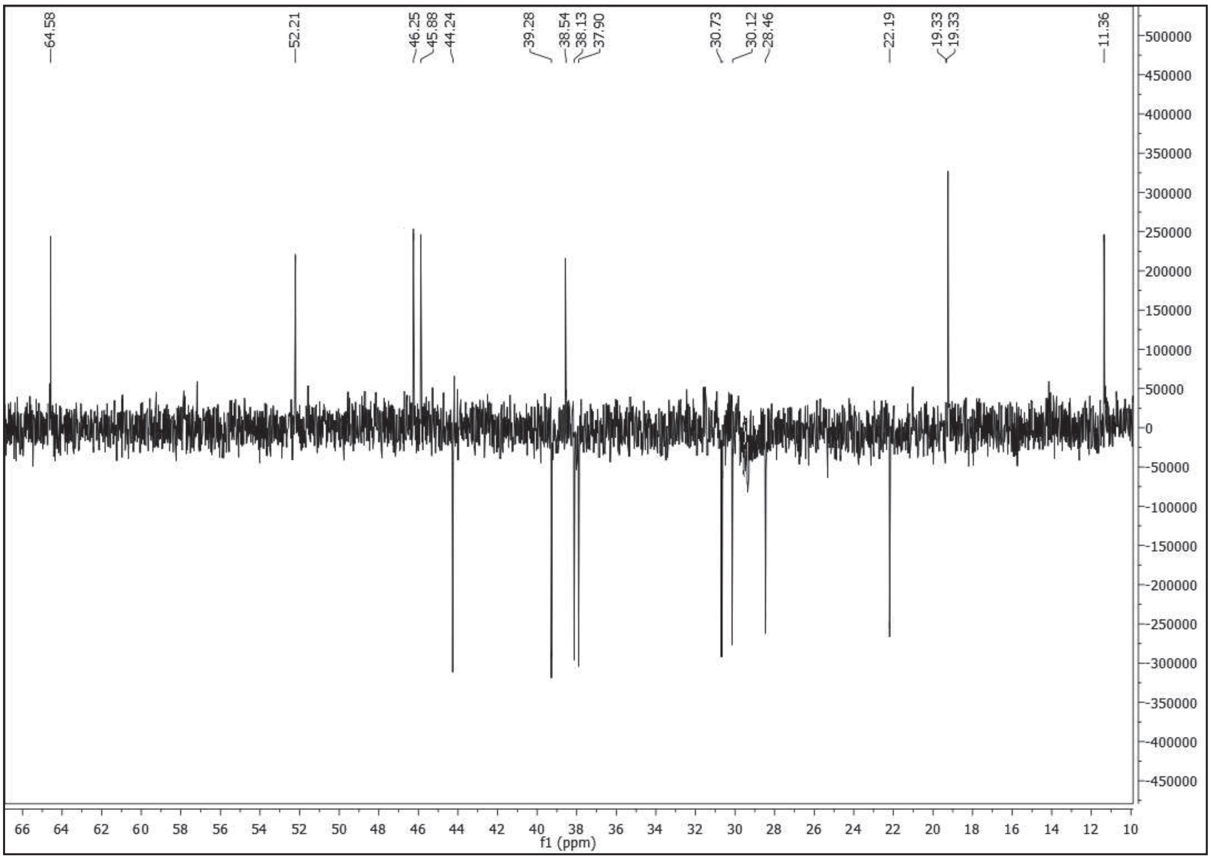

S21. DEPT of 16α-hydroxy-17a-oxa-D-homo-5α-androstan-3,17-dione (**13**)

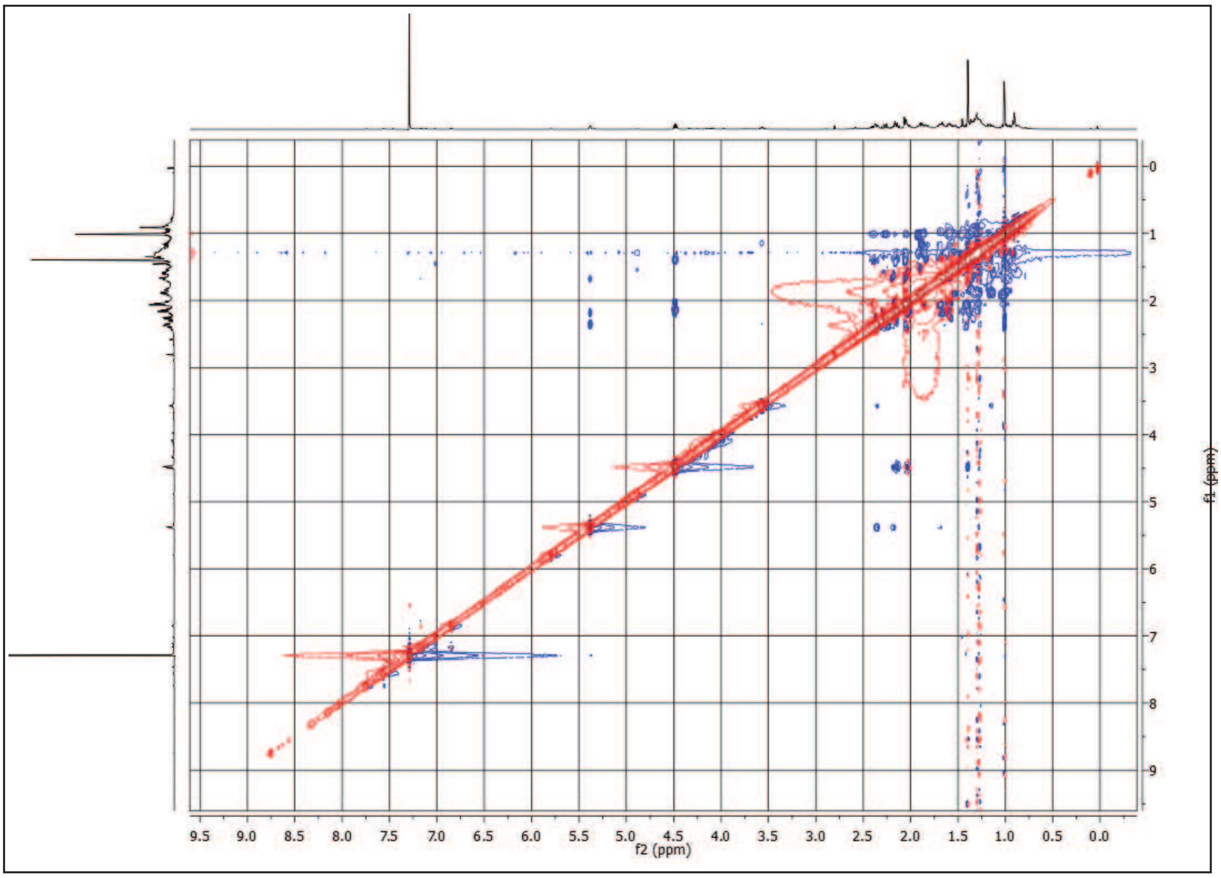

S22. NOESY spectrum of 16α-hydroxy-17a-oxa-D-homo-5α-androstan-3,17-dione (**13**)

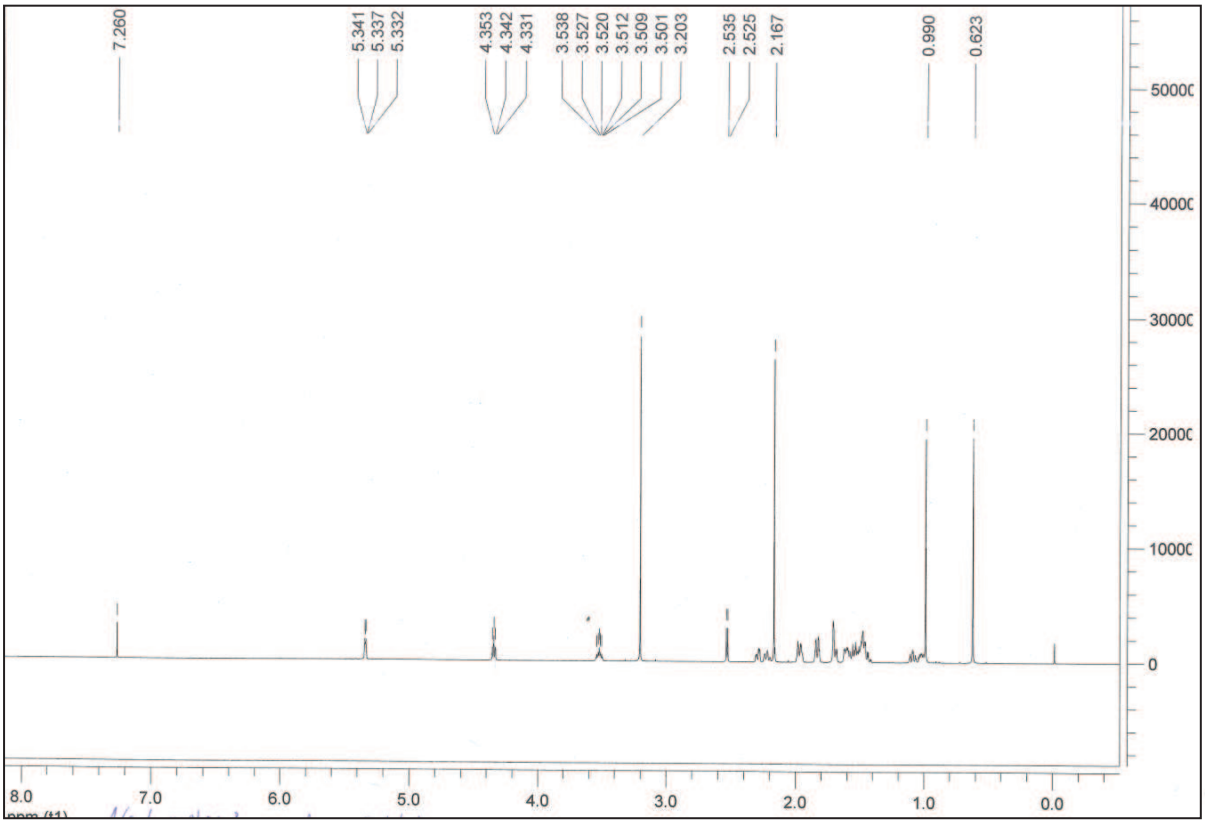

S23. ¹H NMR spectrum of 16α-methoxy-pregnenolone (**16**).

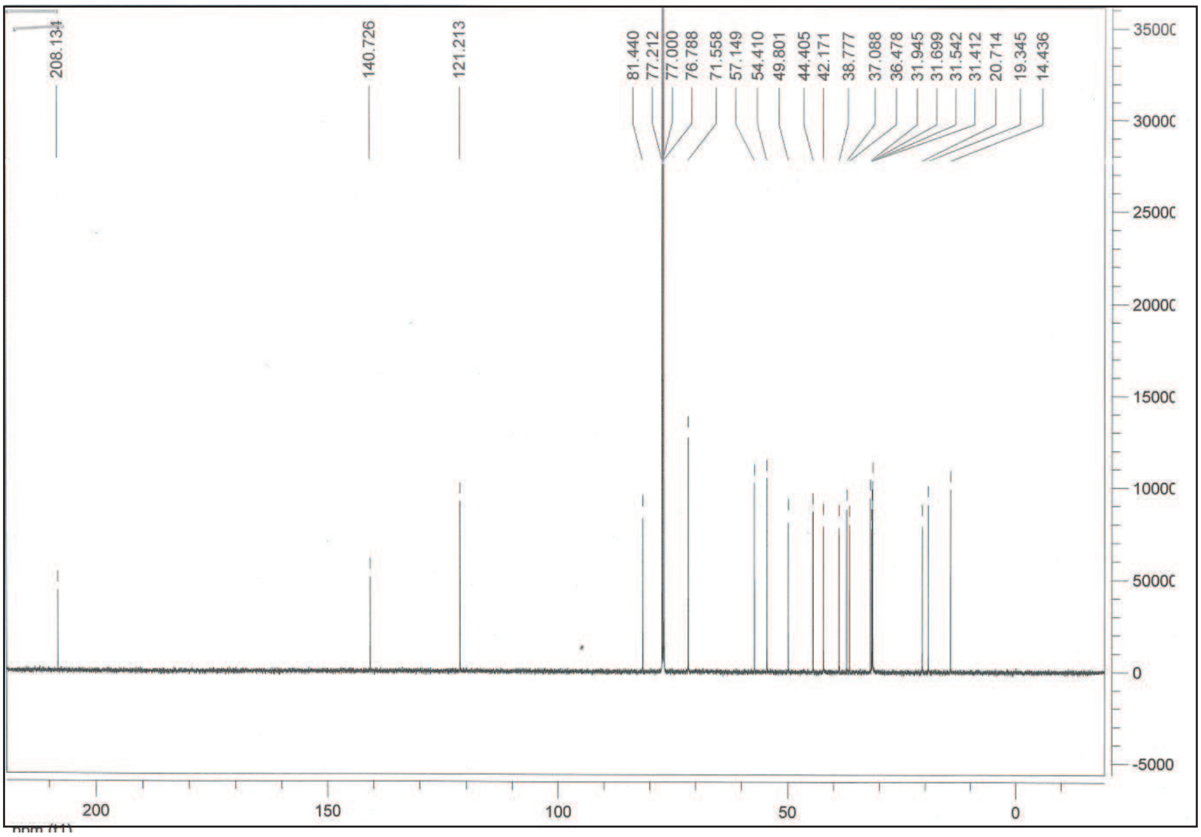

S24. ¹³C NMR spectrum of 16α-methoxy-pregnenolone (**16**).

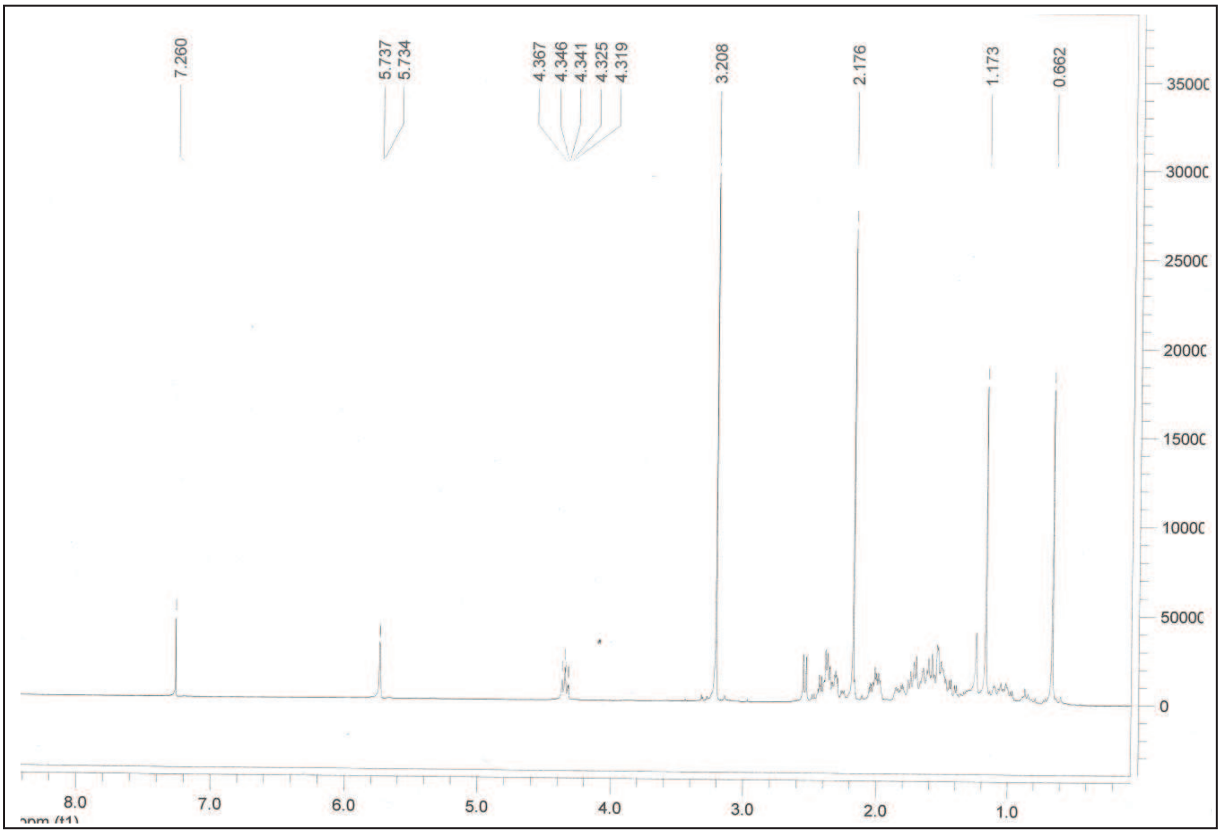

S25. ^{1}H NMR spectrum of 16α-methoxy-progesterone (**17**).

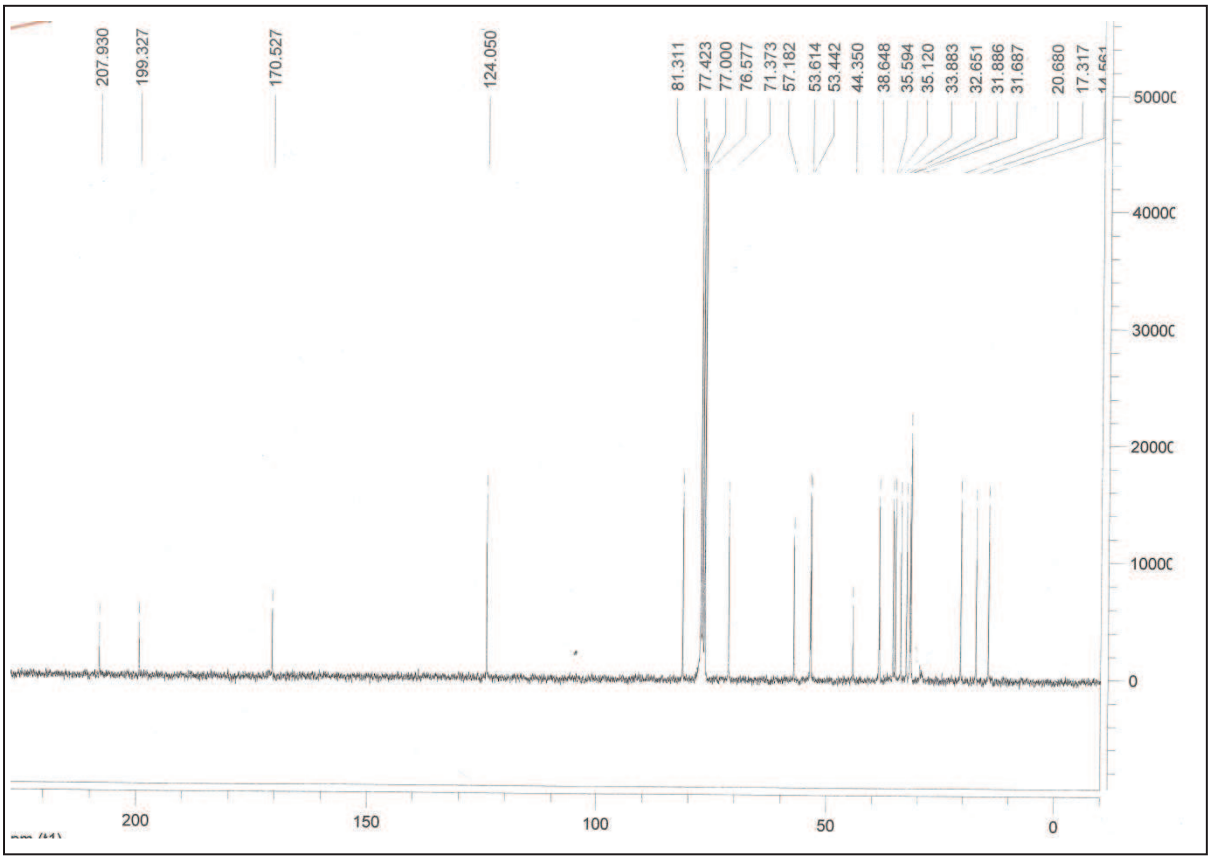

S26. ^{13}C NMR spectrum of 16α-methoxy-progesterone (**17**).

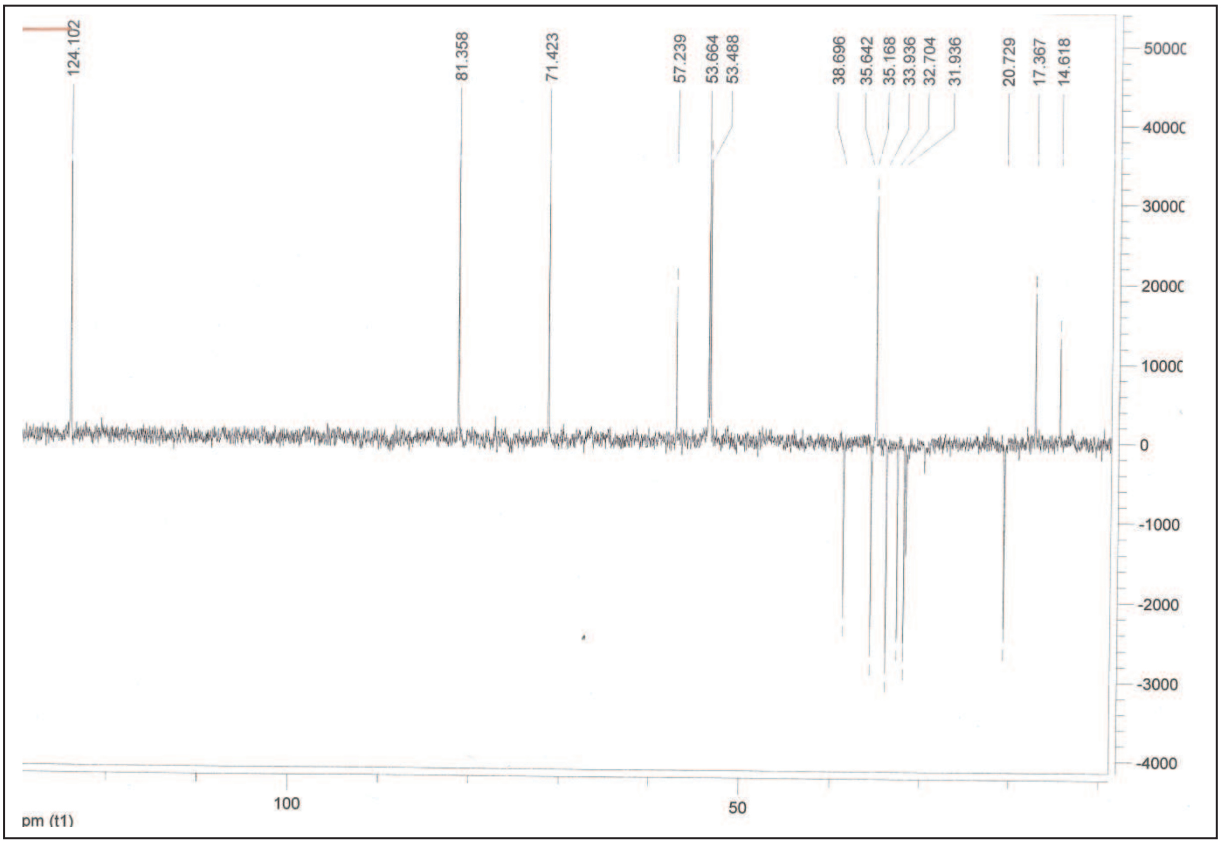

S27. DEPT spectrum of 16α-methoxy-progesterone (**17**).

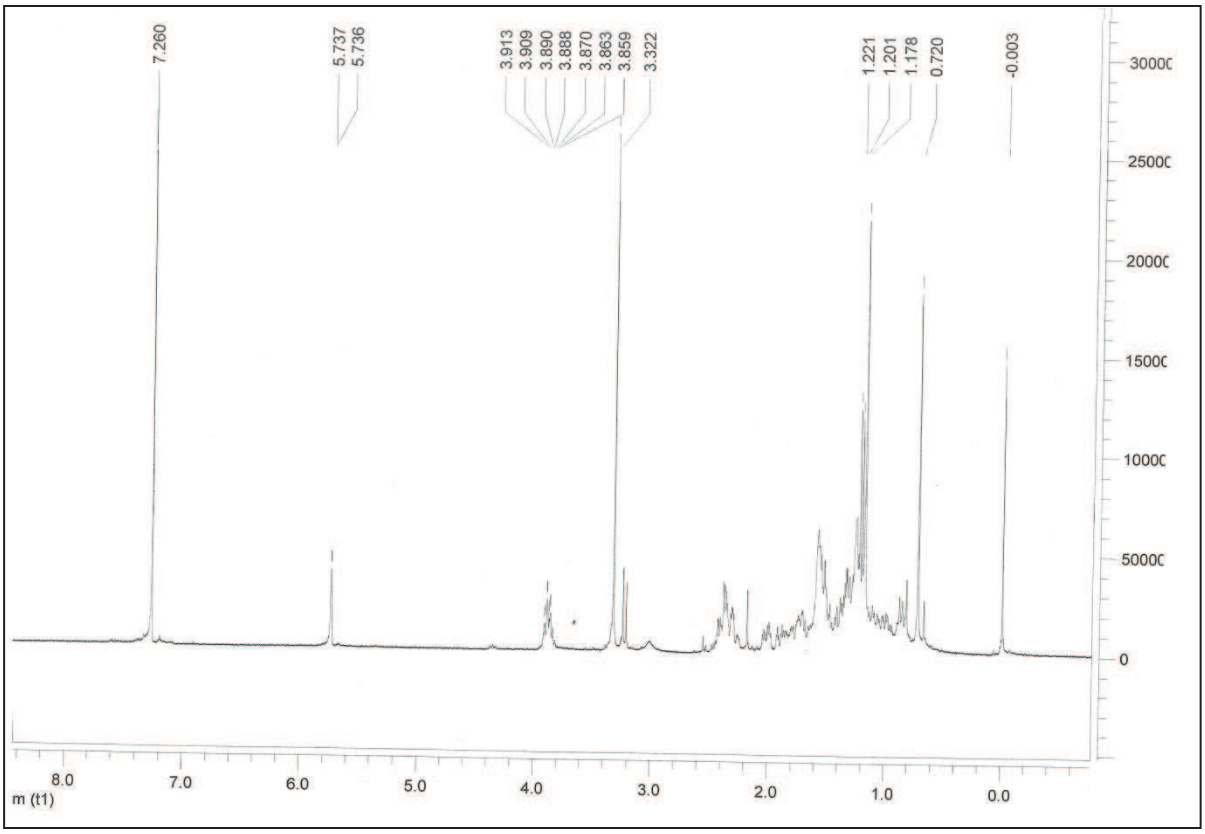

S28. ^{1}H NMR spectrum of (20*R*)-20-hydroxy-16α-methoxy-pregn-4-en-3-one (**18**)

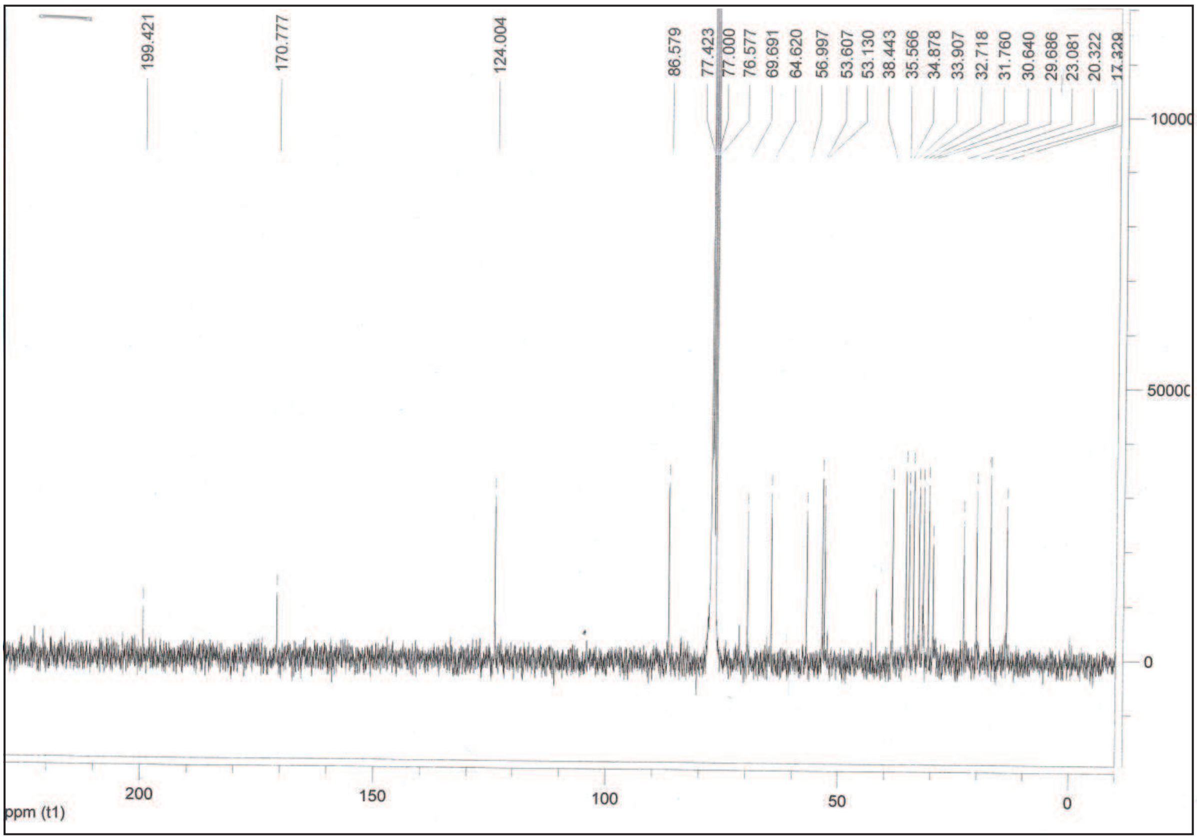

S29. ^{13}C NMR spectrum of (20*R*)-20-hydroxy-16α-methoxy-pregn-4-en-3-one (**18**)

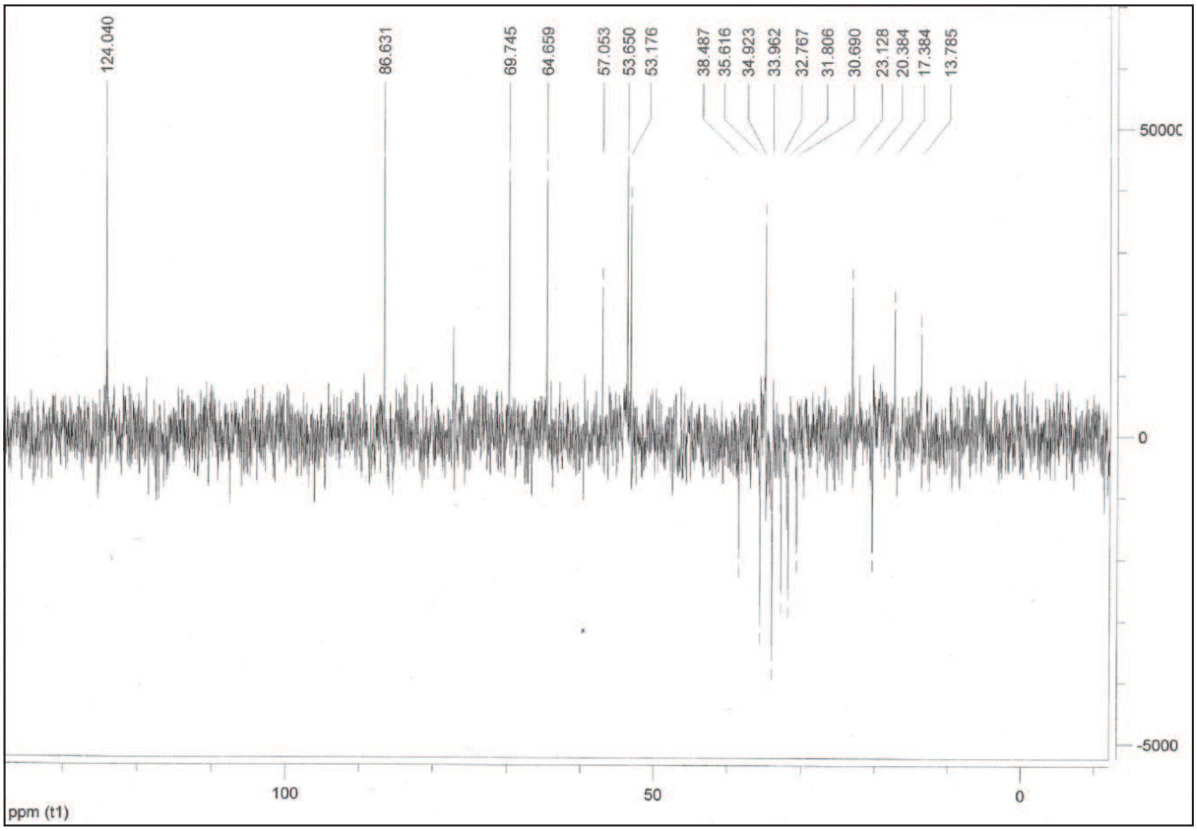

S30. DEPT spectrum of (20*R*)-20-hydroxy-16α-methoxy-pregn-4-en-3-one (**18**)

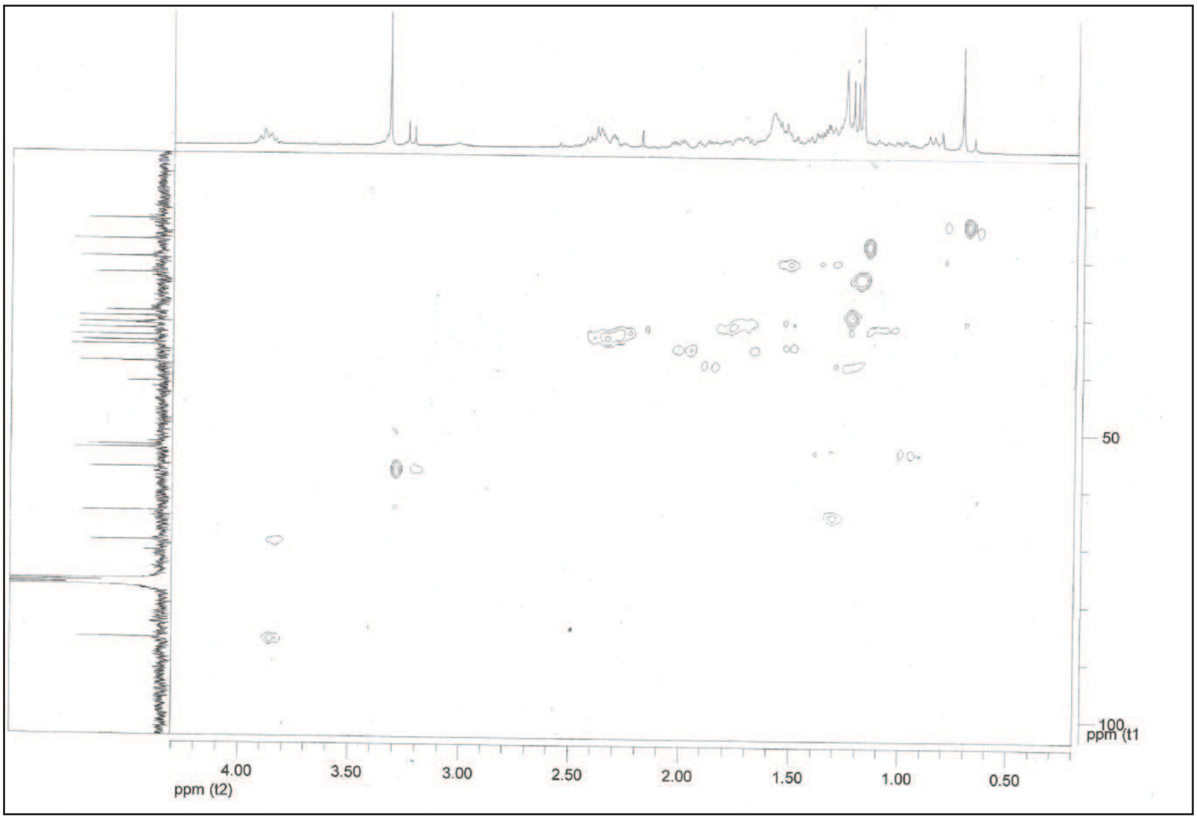

S31. HSQC spectrum of (20*R*)-20-hydroxy-16α-methoxy-pregn-4-en-3-one (**18**)

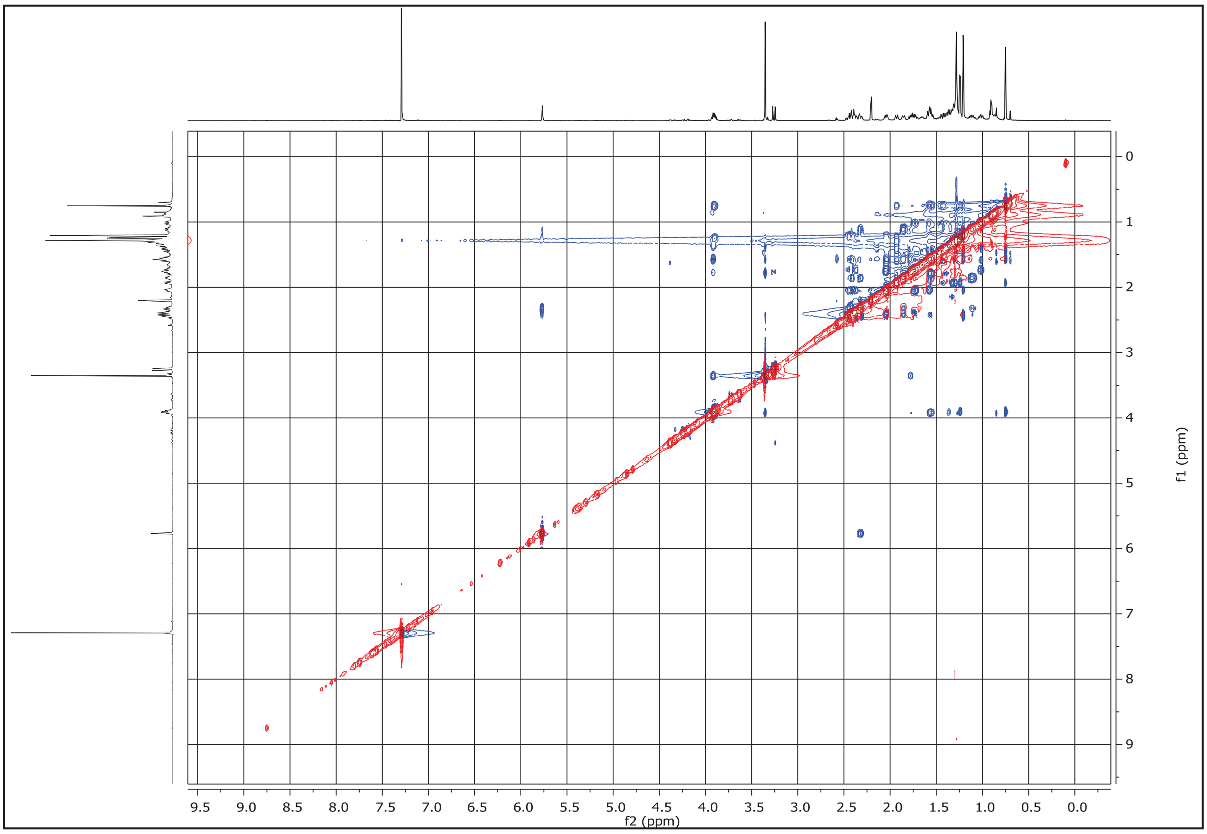

S32. NOESY spectrum of (20*R*)-20-hydroxy-16α-methoxy-pregn-4-en-3-one (**18**)

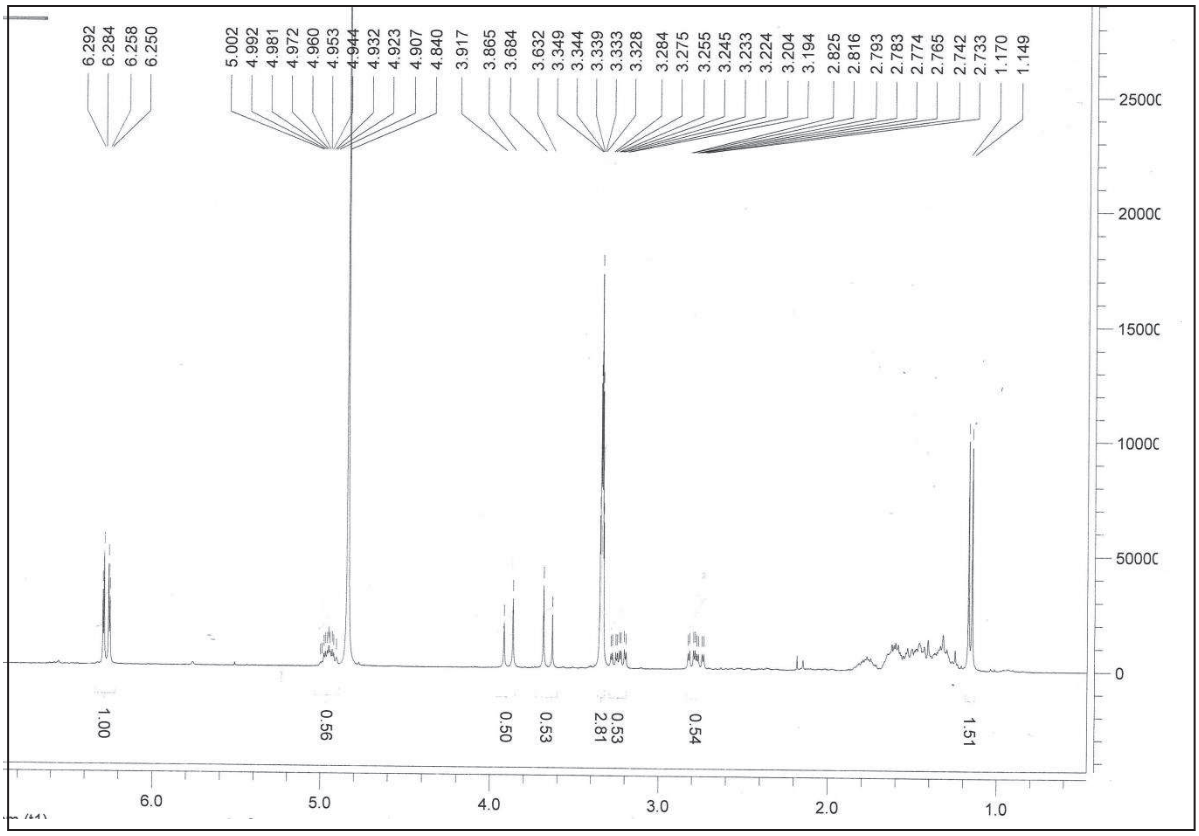

S33. ^{1}H NMR spectrum of (*S*)-curvularin (**19**)

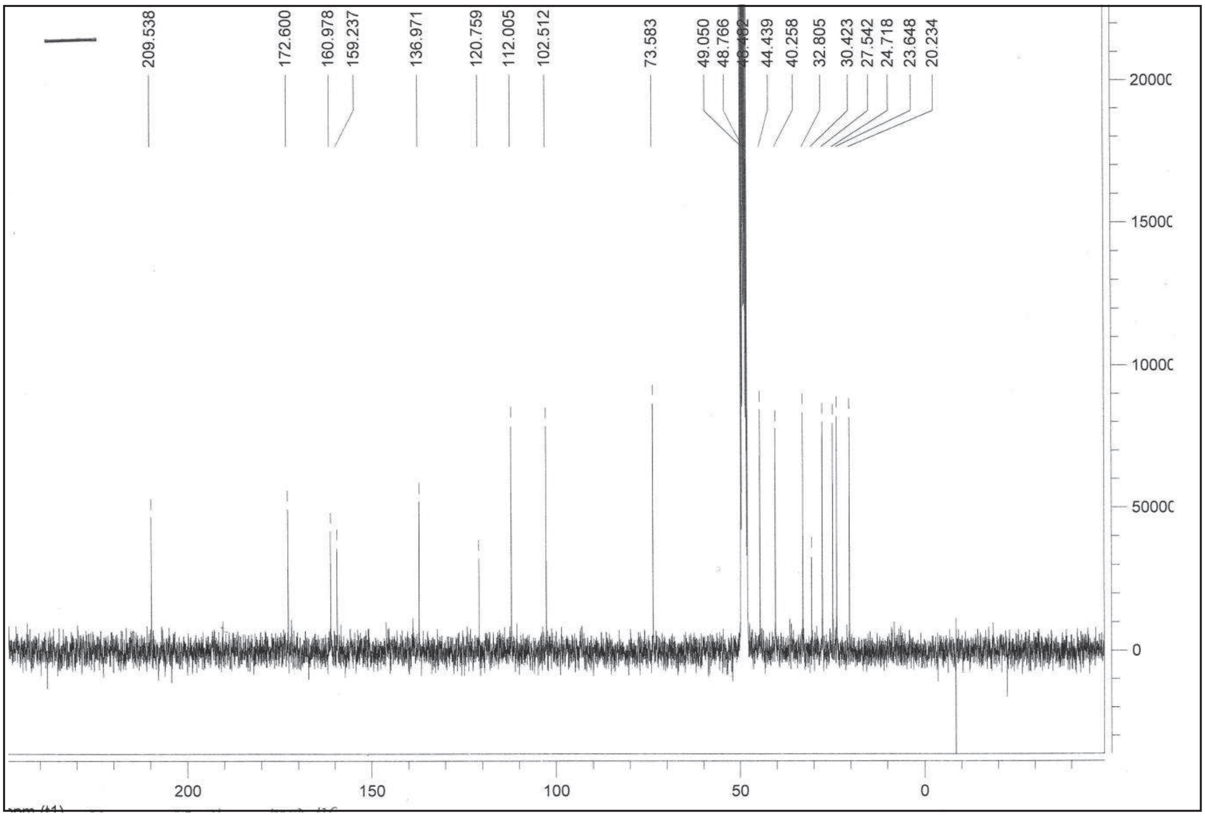

S34. ^{13}C NMR spectrum of (*S*)-curvularin (**19**)
